# Gene Duplication and Gain in the Trematode *Atriophallophorus winterbourni* Contributes to Adaptation to Parasitism

**DOI:** 10.1093/gbe/evab010

**Published:** 2021-01-23

**Authors:** Natalia Zajac, Stefan Zoller, Katri Seppälä, David Moi, Christophe Dessimoz, Jukka Jokela, Hanna Hartikainen, Natasha Glover

**Affiliations:** 1 Eawag, Swiss Federal Institute of Aquatic Science and Technology, Dübendorf, Switzerland; 2 ETH Zurich, Department of Environmental Systems Science, Institute of Integrative Biology, Zurich, Switzerland; 3 Research Department for Limnology, University of Innsbruck, Mondsee, Austria; 4 Department of Computational Biology, University of Lausanne, Switzerland; 5 Swiss Institute of Bioinformatics, Lausanne, Switzerland; 6 Center for Integrative Genomics, Lausanne, Switzerland; 7 Centre for Life’s Origins and Evolution, Department of Genetics Evolution and Environment, University College London, United Kingdom; 8 Department of Computer Science, University College London, United Kingdom; 9 School of Life Sciences, University of Nottingham, University Park, United Kingdom

**Keywords:** comparative genomics, evolution, phylogeny, selection

## Abstract

Gene duplications and novel genes have been shown to play a major role in helminth adaptation to a parasitic lifestyle because they provide the novelty necessary for adaptation to a changing environment, such as living in multiple hosts. Here we present the de novo sequenced and annotated genome of the parasitic trematode *Atriophallophorus winterbourni* and its comparative genomic analysis to other major parasitic trematodes. First, we reconstructed the species phylogeny, and dated the split of *A. winterbourni* from the Opisthorchiata suborder to approximately 237.4 Ma (±120.4 Myr). We then addressed the question of which expanded gene families and gained genes are potentially involved in adaptation to parasitism. To do this, we used hierarchical orthologous groups to reconstruct three ancestral genomes on the phylogeny leading to *A. winterbourni* and performed a GO (Gene Ontology) enrichment analysis of the gene composition of each ancestral genome, allowing us to characterize the subsequent genomic changes. Out of the 11,499 genes in the *A. winterbourni* genome, as much as 24% have arisen through duplication events since the speciation of *A. winterbourni* from the Opisthorchiata, and as much as 31.9% appear to be novel, that is, newly acquired. We found 13 gene families in *A. winterbourni* to have had more than ten genes arising through these recent duplications; all of which have functions potentially relating to host behavioral manipulation, host tissue penetration, and hiding from host immunity through antigen presentation. We identified several families with genes evolving under positive selection. Our results provide a valuable resource for future studies on the genomic basis of adaptation to parasitism and point to specific candidate genes putatively involved in antagonistic host–parasite adaptation.

**Significance**

Transition to parasitism has been associated with gene duplication and gain of novel genes for host exploitation, invasion, and escape from host immunity. In our study, we trace gene duplications and gains across a phylogeny from an ancestral trematode genome to our focal species, the newly sequenced trematode *Atriophallophorus winterbourni*. We characterize gene duplications and gains in three ancestral genomes leading to *A. winterbourni* and outline candidate gene families that have recently undergone duplication and are potentially involved in parasitism.

## Introduction

The adoption of a parasitic lifestyle represents a major niche shift that has occurred multiple times across the tree of life (Poulin and Randhawa 2015; Weinstein and Kuris 2016). The similar selective pressures involved in exploiting hosts have resulted in convergent macroevolutionary features, such as a tendency for morphological simplification (O’Malley et al. 2016) and the associated genome compaction, reduction, and streamlining across many parasite lineages (Peyretaillade et al. 2011; Chang et al. 2015; Lu et al. 2019; Slyusarev et al. 2020). At the same time, parts of the parasite genome involved in, for example, host exploitation and life-cycle complexity may have experienced expansions. Comparative genomic analyses have implied that gene duplications can drive innovation in gene function during radiations of parasitic lineages (Zarowiecki and Berriman 2015).

Novel gene functions involved in the response to host immunity may be particularly important for the evolution of parasitism. For example, mucins, a family of heavily glycosylated surface epithelial proteins, have undergone multiple rounds of duplication in the blood fluke, *Schistosoma mansoni.* Mucins frequently recombine, generating antigenic variation through splice variants (Roger et al. 2008). Increased life-cycle complexity, especially within the parasitic flatworms (Poulin and Randhawa 2015), may have also driven the evolution of functional novelty involved in host exploitation strategies. For instance in *S. mansoni*, multiple duplication events in the gene superfamily SCP/TAPS (sperm-coating protein/TPx/antigen 5/pathogenesis-related protein 1) have led to an array of proteins that are now associated with an active role in penetration of the snail host tissues (Cantacessi and Gasser 2012). Duplicated genes, which evolve beyond sequence recognition, can also give rise to lineage-specific genes (“gained” genes), which can confer specific, novel traits, important in adaptation of that lineage to its particular niche (David et al. 2008; Takeuchi et al. 2016).

With the whole genome sequences of over 30 nematodes (roundworms) and 25 platyhelminth (flatworms, including trematodes) species, it has been possible to characterize the births and expansions of new gene families arising by duplication at key taxonomic levels (Rödelsperger 2018). Nematodes and platyhelminths are two invertebrate animal phyla consisting of parasitic and free-living organisms with the parasitic ones causing major animal, crop, and human diseases, as well as being a major economic burden (Disease and Injury Incidence and Prevalence Collaborators, GBD 2016; International Helminth Genomes Consortium 2019).

**Fig. 1:**
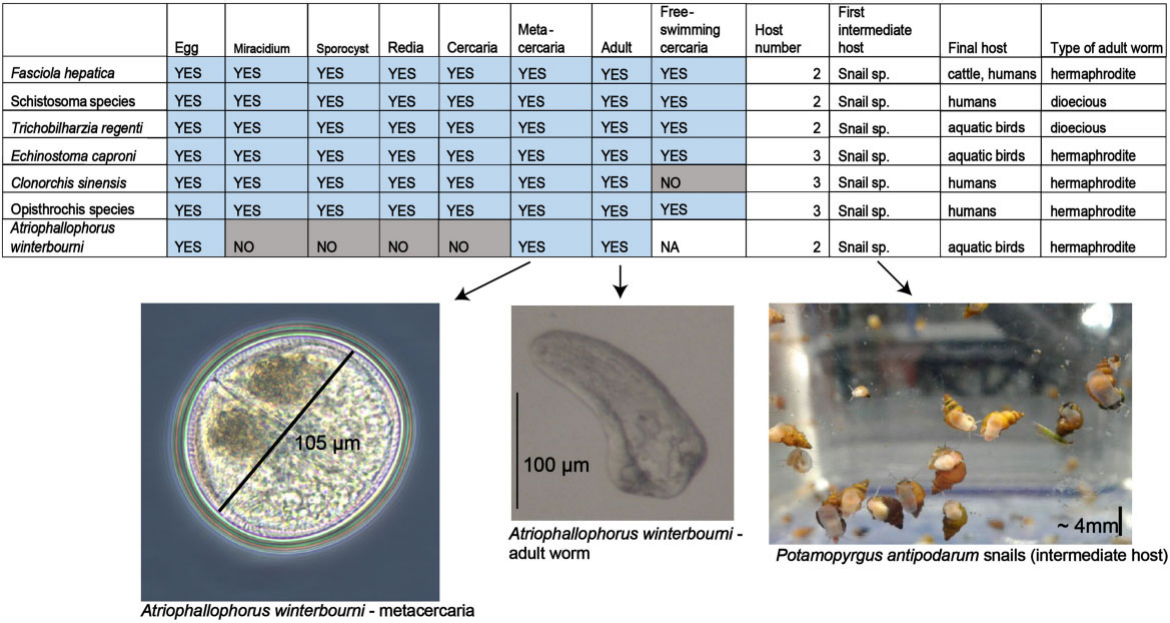
A summary table showing several shared life cycle characteristics of the trematodes used in the study. The first seven columns indicate the presence (blue) or absence (gray) of developmental stages in each parasite’s life cycle. “Host number” indicates the number of hosts in a parasite’s life cycle, “Type of adult worm” indicates whether the adult worms in the final host are hermaphroditic or dioecious (both males and females present). Species within the genera *Schistosoma* and *Opisthorchis* are grouped due to identical characteristics. The photographs below represent the metacercaria and adult stage of *A. winterbourni* and the intermediate host of *A. winterbourni* (*P. antipodarum* snail) (photographs taken by N. Zajac and K. Seppälä).

The microphallid *Atriophallophorus winterbourni* (syn. *Microphallus* sp. or *Microphallus livelyi*) is a digenean trematode parasite native to the lakes of New Zealand (Blasco-Costa et al. 2020). It alternates between two hosts in its life cycle; the intermediate host is *Potamopyrgus antipodarum*, a prosobranch dioecious mud snail (Warwick 1952; Winterbourn 1970) and the final hosts are waterfowl, mainly dabbling ducks (Lively and McKenzie 1991). Multihost life cycle is a general characteristic of all digenean trematodes, and always includes a molluscan species as an intermediate host and a vertebrate as the final host (Galaktionov and Dobrovolskij 2003) (supplementary box S1, Supplementary Material online). The metacercarial asexual stage of *A. winterbourni* develops in the gonad of the snail, which is consequently castrated. The adult worm stage occurs in the gut of waterfowl, where the worms reproduce sexually, producing eggs released with the waterfowl feces (Lively and McKenzie 1991). *Atriophallophorus winterbourni* notably lacks several life cycle stages known to occur in other digenean trematodes, including sporocyst, redia, cercaria, and possibly miracidia stages (fig. 1). Unlike some other well-studied digenean trematodes (see fig. 1 and supplementary box S1, Supplementary Material online), *A. winterbourni* is not known to infect humans and has low virulence in its final bird host. The *Potamopyrgus–Atriophallophorus* system has been studied intensively because the parasite seems to be in a tight coevolutionary relationship with its host in natural populations (Lively et al. 2004). The host–parasite interaction has been used to test alternative explanations for the maintenance of sex in *Potamopyrgus* snails (Lively 1987, 1989). Previous field and laboratory studies suggest that *A. winterbourni* adaptation to local host populations is genotype specific to a degree that the parasite population can adapt to specifically infect the most common host genotypes, which creates negative frequency-dependent dynamics between the two (Dybdahl and Lively 1996; Lively et al. 2004; Jokela et al. 2009). Additionally, recent experimental evidence has indicated that the parasite alters the behavior of the snail, causing it to migrate to the shallow parts of the lake where the final host resides (Feijen F., Buser C., Klappert K., Kopp K., Lively C., Zajac N., Jokela J., in preparation).

In this study, we assembled de novo the *A. winterbourni* reference genome, annotated protein-coding genes, and assigned putative functions using Gene Ontology (GO). With the knowledge from previous studies of pathways and gene families potentially important in trematode adaptation to parasitism, we used comparative genomics to contrast *A. winterbourni* with other trematodes. We studied the evolution of homologous gene families across the phylogeny of platyhelminths using hierarchical orthologous groups (HOGs), or sets of orthologs/paralogs which all originate from a single gene in the last common ancestor of a clade of interest (Altenhoff et al. 2013). By tracing HOGs along the species tree, it is possible to infer the evolutionary history of gene loss, gain, and duplications since the ancestral gene. Using HOGs, we reconstructed the ancestral digenean trematode genome, the Plagiorchiida ancestral genome, and the ancestral genome before the split of Xiphidiata and Opisthorchiata suborders. Using these ancestral genomes, we identified the evolutionary events (duplications, gains, and retention of 1:1 orthologs) that shaped each gene family in the lineage leading to *A. winterbourni.* We characterized the duplicated, gained, and 1:1 orthologs (i.e., conserved/retained) genes shared between all trematodes, as well as those specific to *A. winterbourni.* We discuss the relevance and function of these gene families in *A. winterbourni* and search for signatures of positive selection in two of the largest gene families. We use the inferred changes in the gene content to better understand the genetic novelty necessary for adaptation to parasitic lifestyles in the lineage leading to *A. winterbourni.* Through outlining candidate genes for parasitism, we provide a basis for future studies on the genomics of parasite–host coevolution and we broaden the knowledge on trematode evolutionary history.

## Materials and Methods

### Parasite Collection and DNA Extraction

*Potamopyrgus antipodarum* snails infected with *A. winterbourni* were collected from Lake Alexandrina (New Zealand, South Island) in January 2017 from several shallow localities (<1.5 m) by pushing a kicknet through the vegetation. The snails were transported to the Swiss Federal Institute of Aquatic Science (Eawag, Dübendorf, Switzerland) within 2 weeks of collections and were kept in boxes of 500 snails in a flow-through system that filtered the water every 12 h. Snails were fed spirulina ad libitum (*Arthospira platensis*, Spirulina California, Earthrise) once a day.

Infected snails were individually dissected and 200–1,000 *A. winterbourni* metacercariae were isolated under 10×–20× magnification. The metacercariae were hatched into adult worms (see supplementary methods S1, Supplementary Material online for details). Obtaining adult worms was necessary to separate the parasite from the double-walled metacercarial cyst that contained both the parasite and the snail DNA (Galaktionov and Dobrovolskij 2003). The worms were lysed using a CTAB buffer and Proteinase K (2 mg/ml) with overnight incubation at 55 °C (Yap and Thompson 1987). DNA was isolated using a chloroform: isoamyl alcohol solution (24:1) and precipitated with sodium acetate (3 M). The resulting pellet was washed twice with 70% ethanol. DNA was stored in RNase/DNase-free water (Sigma-Aldrich, MO) at −20 °C until sequencing library preparation.

### Estimation of Genome Size

To guide the de novo genome assembly, genome size was estimated using flow cytometry with Propidium Iodide staining (CyFlow Space, Sysmex). *Atriophallophorus winterbourni* worms were hatched according to the above-described protocol. A pool of 15 worms was stained for 1 h with Propidium Iodide (according to the Partec protocol of CyStain PI Absolute T kit) and treated with DNase-free RNase. Three batches of 15 worms were measured, each taken from a different snail host. The DNA content of 2C nuclei was calculated using heads of isoline *Drosophila melanogaster* males and a laboratory clone of *Daphnia galeata* as two independent standards. For the haploid DNA content of *Drosophila melanogaster*, a value of 175 Mb (Bennett et al. 2003) was used and for *Daphnia galeata* a value of 158 Mb (S. Dennis, personal communication, December 12, 2019). Each standard was run separately with each batch of worms.

### Sequencing

The DNA of *A. winterbourni* was sequenced using Illumina and Pacific Biosciences Technologies (Ambardar et al. 2016). For Illumina sequencing, two infected snails were selected from a shallow water habitat from one site sampled at Lake Alexandrina. A total of 200 ng DNA was extracted from approximately 800–1,000 worms and was sent to the Functional Genomics Center Zurich (University of Zurich, Zurich) for library preparation and paired end sequencing using the Illumina HiSeq4000 sequencing platform. A single TruSeq library was constructed from the DNA using the TruSeq Nano DNA library prep kit according to Illumina protocols, obtaining an average of 500 bp insert size. The library was sequenced without indexing on a single Illumina lane. For Pacific Biosciences sequencing, we selected 33 infected snails from two different sites from a shallow water habitat with a high infection prevalence within Lake Alexandrina. We assumed no distinct or significant population structure for the parasite from different sites within the same habitat zone, as previously shown for the snail host (Paczesniak et al. 2013). Genomic material was isolated from a pool of approximately 13,000–30,000 worms. The high-molecular weight DNA with an average length of 45,000 bp (assessed with a Bioanalyser) was sent for sequencing to the Functional Genomics Center Zurich (University of Zurich, Zurich), where it was sequenced with the Pacific Biosciences RSII sequencing platform. A 10-kb SMRT-bell library was constructed from a total of 10 µg of DNA. The library was sequenced using three SMRT cells using P6/C4 chemistry. Primary filtering was performed by Functional Genomics using the SMRT Link software from Pacific Biosciences. We performed secondary filtering, choosing only reads of at least 1,000 bp in length and with read quality >80%. No error correction was performed on the PacBio data at this stage, as it was corrected later with the Illumina data during the hybrid assembly.

### Illumina Data Correction

A quality trimming step was performed with Trimmomatic 0.35 on the raw Illumina HiSeq data before proceeding with the assembly. Adapter sequences were removed and bases with a phred quality score below 5 were removed from the start and the end of the reads. Reads were scanned with a sliding window of 4 and were clipped if the average quality per base dropped below 15. Reads shorter than 50 bp were discarded. The reads were then submitted to PRINSEQ (Schmieder and Edwards 2011) for filtering for ambiguous bases (Ns), characters different than A, C, G, T or N, and for removal of exact duplicates. For assessment of contamination, we used taxonomic interrogation of the paired reads with Kraken v2, standard database (Wood and Salzberg 2014).

### Hybrid Assembly and Annotation

Paired reads from Illumina were used together with long reads from Pacific Biosciences for a hybrid assembly with the MaSuRCA 3.2.3 assembler using default parameters (Zimin et al. 2013). Redundans 0.13c (Pryszcz and Gabaldón 2016) and AGOUTI (Zhang et al. 2016) were used for improvements. Redundans improves the quality of the assembly by reduction, scaffolding, and gap closing (Pryszcz and Gabaldón 2016). The reduction steps consist of identification and removal of heterozygous contigs, based on pairwise sequence similarity searches. Heterozygous contigs are expected to have high sequence identity (Pryszcz and Gabaldón 2016). The quality was assessed using the N50 statistic, BUSCO 3.0.2 (Benchmarking Universal Single Copy Orthologs) (Waterhouse et al. 2018), and Blobtools 0.9.19.5 (Laetsch and Blaxter 2017). BUSCO 3.0.2 assesses the completeness of single copy orthologs based on evolutionary-informed expectations about gene content using the lineage data set metazoa_odb9. Blobtools 0.9.19.5 was used for taxonomic partitioning of the assembly. All scaffolds >50,000 bp (2,718 scaffolds) plus a random sample of scaffolds <50,000 bp from the assembly (2,661 scaffolds) were submitted to BLAST 2.3.0 using the NCBI nr database for taxonomic annotation. Taxonomic assessment of those scaffolds was used as input for Blobtools. The paired and filtered Illumina reads and PacBio reads of at least 1,000 bp in length and with read quality >80% were mapped back to the final assembly with BWA-MEM 0.7.17, yielding an average of 143× coverage per base (125× from the Illumina reads and 18× from the PacBio reads).

Genome annotation was performed using the Maker 2.31.9 annotation pipeline (for details see supplementary methods S2, Supplementary Material online) (Cantarel et al. 2008). The completeness and quality of the annotation was assessed with BUSCO and with full-length transcript analysis using BLAST+ (see supplementary methods S3, Supplementary Material online). GO annotation of the coding sequences was performed with Pannzer2 (Törönen et al. 2018), EggNOG (Diamond mapping mode) (Huerta-Cepas et al. 2016) and OMA (“Orthologous MAtrix”) (Altenhoff et al. 2018) web browsers with each data set used separately for GO enrichment analyses (http://ekhidna2.biocenter.helsinki.fi/sanspanz/ [last accessed: October 2019], http://eggnogdb.embl.de/#/app/home [last accessed: January 2020], https://omabrowser.org/oma/functions/ [last accessed: December 2019]). We also assessed the percentage of all GO terms annotated in *A. winterbourni* with experimental evidence in nematode or trematode (supplementary methods S4, Supplementary Material online).

### Comparative Genomics and Ancestral Genome Reconstruction

**Fig. 2:**
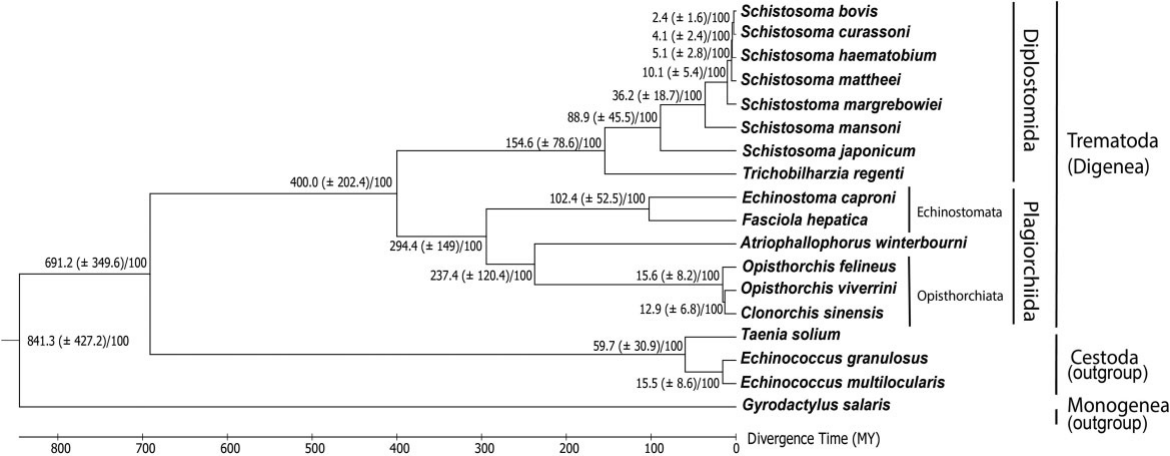
Phylogenetic tree and classification of species used in the analysis. The data used for the tree were all Orthologous Groups from the OMA analysis with genes from at least 15 species present (238 groups of orthologs). The combined data was used in IQ-TREE to create a robust consensus species tree. The tree and the combined alignment of 238 groups of orthologs was used in Mega-X 6.06 for reconstruction of the time tree. The scale below indicates divergence time in million years (Myr). Each node has a divergence time with the confidence interval indicated in brackets in million years and a bootstrap support indicated after a slash.

We selected 20 species of platyhelminthes and nematodes for comparative genomic analysis. The choice of both nematodes and trematodes was based on their comparisons in other helminth genomic analyses (Zarowiecki and Berriman 2015; International Helminth Genomes Consortium 2019) and will allow for future comparison of trematodes to model species of nematodes. The species consisted of 14 digenean trematodes (including our focal species), 3 species of parasitic cestodes, 1 species of parasitic monogeneans, and 2 species of free-living nematodes (see supplementary box S1, Supplementary Material online, fig. 2). We chose these species on the basis of close relatedness to *A. winterbourni* and quality of the genome assembly and annotation (species also used in International Helminth Genomes Consortium [2019]). The proteomic, genomic, and transcriptomic sequences for analysis were obtained from the NCBI database of invertebrate genomes (ftp.ncbi.nlm.nih.gov) and from the EBI database (ftp://ftp.ebi.ac.uk/). For the analysis, we used the most recent genomes from those databases with available transcriptomic data (CDS_genomic) and protein annotation (see supplementary table S1, Supplementary Material online).

The OMA standalone (Orthologous Matrix) software was used for inference of HOGs of genes shared between species (Altenhoff et al. 2019). This software conducts an all-against-all comparison to identify the evolutionary relationships between all pairs of proteins included in the custom-made database of the 20 genomes. The program was run with default parameters and with the “bottom-up” algorithm for inference of HOGs. *Caeorhabditis elegans* and *P. pacificus* were specified as outgroup species. After obtaining the phylogenetic species tree (see next section), OMA was rerun with the precise species tree specified.

The data obtained from OMA was then analyzed with the python library pyHam (Train et al. 2019). With pyHam we reconstructed a model of the ancestral genomes at each stage of the phylogeny leading to *A. winterbourni* and carried out all comparisons between ancestral and extant genomes to obtain classes of duplicated, gained, retained, or lost genes (see Jupyter notebook supplementary material S6, Supplementary Material online). We also used pyHam to visualize genomic changes along each branch of the phylogenetic tree.

### Phylogenetic Species Tree

OMA Groups, that is, Orthologous Groups, from the OMA output were used for phylogenetic tree construction, as they are stringent groups of orthologs and do not contain paralogs (Zahn-Zabal, Dessimoz, Glover 2020). The phylogenetic tree was constructed following the protocol of Dylus et al. (2020). Briefly, Orthologous Groups containing at least 15 species of monogeneans, cestodes, and trematodes were extracted using the custom script filter_groups.py from the git repository: https://github.com/DessimozLab/f1000_PhylogeneticTree. Nematodes were excluded from precise phylogenetic and time tree reconstruction, as they are too evolutionarily distant. Within each Orthologous Group, sequences were aligned using MAFFT (mafft 7.273, 1,000 cycles of iterative refinement) (Katoh et al. 2009). The separate alignments were concatenated into one supermatrix using a custom script concat_alignment.py from the git repository: https://github.com/DessimozLab/f1000_PhylogeneticTree. The final size of the supermatrix was 145,802 sites for all 18 species. No columns from the supermatrix were excluded. The supermatrix was used as input for IQ-TREE maximum-likelihood phylogenetic tree construction (Trifinopoulos et al. 2016; Kalyaanamoorthy et al. 2017; Hoang et al. 2018) using the ModelFinder Plus option for finding the best fitting model. Branch support was calculated with 1,000 Ultrafast bootstrap alignments and 1,000 iterations. The maximum-likelihood tree was confirmed with ASTRAL III (Zhang et al. 2018) by constructing a species tree from gene trees of the 238 Orthologous Groups. Each gene tree was first constructed with IQ-TREE using ModelFinder Plus for choosing an appropriate model; branch support was calculated with 1,000 bootstrap alignments and 1,000 iterations. The IQ-TREE tree, together with the supermatrix, were used in Mega-X 6.06 for time tree reconstruction using the Maximum-Likelihood RelTime method (Tamura et al. 2013). We used two pieces of evidence for time calibration, discussed in the Results.

### GO Enrichment Analysis

We performed GO annotation for each species using Pannzer2, EggNOG (Diamond mapping mode) and OMA (Huerta-Cepas et al. 2016; Altenhoff et al. 2018; Törönen et al. 2018). Each extant species genome was functionally annotated with orthology-informed putative functions using OMA, Pannzer2, and EggNOG reaching between 26% and 96% of genes annotated for each species (supplementary table S2, Supplementary Material online). We then performed GO enrichment analysis using GOATOOLS (Klopfenstein et al. 2018), which finds statistically over- and under-represented GO terms in the set of genes of interest compared with all the GO terms in the background population. For analyses that were species specific, the background set was all the genes in the genome. For analyses of ancestral genomes, the background population was all the ancestral genes, that is, the set of HOGs at that taxonomic level. To get the GO terms for any particular ancestral gene/HOG, we took the union of all the GO terms in the extant “children” species. Fisher’s exact test was used for computing uncorrected *P* values. The *P* values were then corrected using the Bonferroni method and retained if the corrected *P* value was <0.05. Subsequently, all enriched GO terms were categorized into GO slim categories using the AGR subset (Alliance of Genome Resources, http://geneontology.org/docs/download-ontology/, last accessed: May 7, 2020) and unique genes within each enriched GO slim category were counted. For each GO term, the IC (Information Content) score was calculated as: IC(*t*) = −log(*P*(*t*)) with *P*(*t*) being estimated as the empirical frequency of the term in the UniProt-GOA database (Barrell et al. 2009). The average IC was calculated for each GO slim term using the IC values of all enriched GO terms in each category (Mistry and Pavlidis 2008; Mazandu and Mulder 2014). GO slim terms were used in summarizing the data.

### Estimation of d*N*/d*S* in Gene Families in *Atriophallophorus winterbourni*

HOGs 25969 and 36190 with over 30 *A. winterbourni* genes were investigated for signatures of positive selection. All proteins within the two families were submitted to NCBI BLASTP to find their best hit against the nr database and obtain putative functions. We then applied the protocol from Jeffares et al. (2015) to estimate the nonsynonymous to synonymous substitution rate ratio within each HOG and to investigate whether selection models explain the data better than null models (Yang 1997; Kohlhase 2006). Protein sequences were aligned using Clustal Omega (Madeira et al. 2019), then converted to codon alignment in Phylip format with PAL2NAL (Suyama et al. 2006). Positive selection analyses are sensitive to alignment errors; thus the gap-ridden alignment of HOG 36190 was subjected to a more stringent alignment filtering, guided by the approach proposed by Moretti et al. (2014) (for details, see supplementary methods S5, Supplementary Material online). Branch site models in codeml were used to estimate d*N*, d*S*, and *ω* (d*N*/d*S*) (model = 2, NSsites = 2). The likelihood ratio test (LRT) was used to determine significance. Gene trees were constructed with protein sequence alignments using IQ-TREE (Trifinopoulos et al. 2016; Kalyaanamoorthy et al. 2017; Hoang et al. 2018). First an initial parsimony tree was created by a phylogenetic likelihood library; 168 protein models were then tested for best fit with the data according to the Bayesian Information Criterion. Branch support was calculated with 1,000 bootstrap alignments (ultrafast bootstrap) and 1,000 iterations. The models chosen were JTT + F + G4 for HOG 25969 (general matrix with empirical amino acid frequencies from the data and discrete Gamma model with four categories) and WAG + G4 for HOG 36190 (general matrix with discrete Gamma model with four categories).

## Results and Discussion

### Genome of *A. winterbourni*

The de novo sequenced genome of *A. winterbourni* resulted in a final assembly of 601.7 Mb in size, consisting of 26,114 scaffolds with an N50 of 40,108 (see table 1 and supplementary results S1, Supplementary Material online for details). The assembly size was similar to the flow cytometry-based genome size estimate of 550–600 Mb (supplementary fig. S1, Supplementary Material online). The annotation yielded 11,499 predicted protein-coding loci spanning 163.7 Mb, with a mean of 5.8 exons and a median of 4 exons per gene (table 1). The final BUSCO gene set completeness for the annotation was 72% of complete single copy conserved orthologs (see supplementary results S3, Supplementary Material online for protein coding sequence length analysis using BLAST+). Relative to other published trematode genomes, the *A. winterbourni* genome showed good protein sequence length distribution and a comparable BUSCO complete single copy conserved orthologs (supplementary fig. S4, Supplementary Material online, table 1). Functional annotation via GO was successful for 84% of genes using OMA, Pannzer2, and EggNOG (9,674 genes, see supplementary fig. S5 and table S2, Supplementary Material online), with 45.3% of the OMA GO terms and 32% of Pannzer2 GO terms assigned to *A. winterbourni* having experimental evidence in nematodes or trematodes (see supplementary table S2, Supplementary Material online and results S2, Supplementary Material online). In comparison to other Plagiorchiida genomes, the *A. winterbourni* assembly was of similar size and showed similar percentages of noncoding regions, suggesting that no significant genome reduction has occurred in this species (table 1). Transposable elements, interspersed repeats, and low complexity DNA comprised 51.7% of the genome (supplementary table S11, Supplementary Material online). This elevated level of TE content in comparison to closely related Opisthorchiata species (33% *C. sinensis*, 30.3% *O. felineus*, 30.9% *O. viverrini*) (Esch et al. 2002) might be an indication of increased importance of transposable elements in *A. winterbourni* genome evolution.

**Table 1:** Information on the Genome Assemblies Used in the Analysis

Species	Genome Size(Mb)	NB Genes	Scaff. Count	N50	GCContent(%)	BuscoComplete Single(%)	Busco Duplicated(%)	Busco Fragmented(%)	Busco Missing(%)	TotalExon Number	AverageExonLength(bp)	TotalIntron Number	AverageIntronLength (bp)	TotalCoding Sequence(Mb)
*Atriophallophorus winterbourni*	601.7	11,499	26,114	40,108	40.73	56.2	15.7	6.1	22	66,672	233	54,987	1,732	163.7
*Taenia solium*	122	12,467	11,237	68,000	42.9	77.9	1.9	6.7	13.5	69,770	223	57,289	574	48.3
*Echinococcus granulosus*	110.8	11,319	957	712,683	41.7	76.2	2.1	6.5	15.2	75,264	211	63,945	722	62
*Gyrodactylus salaris*	67.4	15,436	6,075	18,400	33.9	67.7	1.5	8.9	21.9	61,693	229	46,257	584	41.1
*Fasciola hepatica*	1,138	14,642	23,604	161,103	44.1	71	1.2	9.5	18.3	83,777	488	72,560	4,168	343.3
*Echinostoma caproni*	834.6	18,607	86,083	27,000	42.5	50.3	1.2	26.9	21.6	65,273	267	46,666	2,451	131.8
*Opisthorchis felienus*	679.25	11,427	13,306	621,022	44.1	53.4	33	4.1	9.5	180,879	261	160,011	3,527	291.9
*Opisthorchis viverrini*	472.26	13,555	16,038	79,767	44	67	0.7	11.6	20.7	59,112	242	48,358	2,734	146.5
*Clonorchis sinensis*	562.7	14,538	2,776	1,628,761	42.6	72.6	1	6.9	19.5	89,304	234	74,766	2,745	226.2
*Trichobilharzia regent*	701.76	22,185	188,369	7,696	37.4	36.4	0.7	35.4	27.5	54,402	277	32,217	1,829	74
*Schistosoma japonicum*	369.9	11,416	1,789	1,093,989	33.8	47.2	34.6	4.3	13.9	130,068	336	113,132	2,372	185.4
*Schistosoma mansoni*	364.5	10,772	885	32,115,376	35.5	71.5	8.6	6.9	13	70,,430	204	57,138	2,475	148.8
*Schistosoma margrebowiei*	367.4	26,189	23,355	35,236	34.3	65.7	1.6	17.4	15.3	79,991	262	53,802	1,925	122.3
*Schistosoma haematobium*	375.89	11,140	29,834	317,484	34.2	71.4	1.6	11.7	15.3	64,235	246	53,148	2,488	148.8
*Schistosoma bovis*	373.4	11,576	4,774	202,989	34.4	68.3	4.3	11.9	15.5	65,265	259	53,689	2,406	146.1
*Schistosoma mattheei*	340.82	22,997	62,061	12,303	34.1	51.3	1.5	24.1	23.1	65,852	263	43,672	1,569	84.1
*Schistosoma curassoni*	344.2	23,546	60,140	13,861	34.2	54.6	1.4	19.6	24.4	69,606	259	46,060	1,576	88.5
*Caeorhabditis elegans*	102.3	20,184	7	17,493,829	35.4	98	0.6	0.8	0.6	285,984	239	250,855	438	63.3
*Echinococcus multilocularis*	115	10,663	1,217	13,800,000	42.2	79.8	2.8	5	12.4	71,022	205	60,677	663	49
*Pristionchus pacificus*	158.5	25,991	47	23,900,000	42.8	91.6	1.3	3.7	22.4	312,244	106	287,319	275	112.2

Note.—For more information, see supplementary table S1, Supplementary Material online. The BUSCO results refer to the protein annotation. The results on exon/intron number and length were calculated from the gff files with genestats script (available at: https://gist.github.com/darencard/fcb32168c243b92734e85c5f8b59a1c3, date accessed July 14, 2020) or obtained from Tsai et al. (2013).

### Species Phylogeny and Molecular Clock

To reconstruct a robust maximum-likelihood phylogenetic tree, 238 Orthologous Groups (groups containing only orthologs, with a maximum one gene per species) shared between at least 15 out of 18 species of Platyhelminths were used. The phylogenetic estimate was well resolved and congruent with previous publications based on genetic markers or whole genomes (fig. 2) (Galaktionov and Dobrovolskij 2003; Lee et al. 2013; International Helminth Genomes Consortium 2019; Blasco-Costa et al. 2020). *Atriophallophorus winterbourni* was placed as sister to the Ophisthorchiata clade with 100% bootstrap support. The time of speciation of *A. winterbourni* from the Opisthorchiata species was estimated to have been 237.4 Ma (±120.4 Myr), that is, during the Carboniferous through the Cretaceous period (fig. 2). The divergence time estimates across the phylogeny were inferred using several independent pieces of evidence, used as calibration points for Time Tree: the existence time of the proto-trematode first associated with a molluscan host around 400 Ma, and the origin of Schistosoma species in the Creataceous period (66–145 Ma) (Gibson 1987; Hausdorf 2000; Blair et al. 2001; Peterson et al. 2004; Parfrey et al. 2011).

### Evolutionary Patterns of Gene 1:1 Orthology, Gain, Loss, and Duplication across Trematoda

**Fig. 3:**
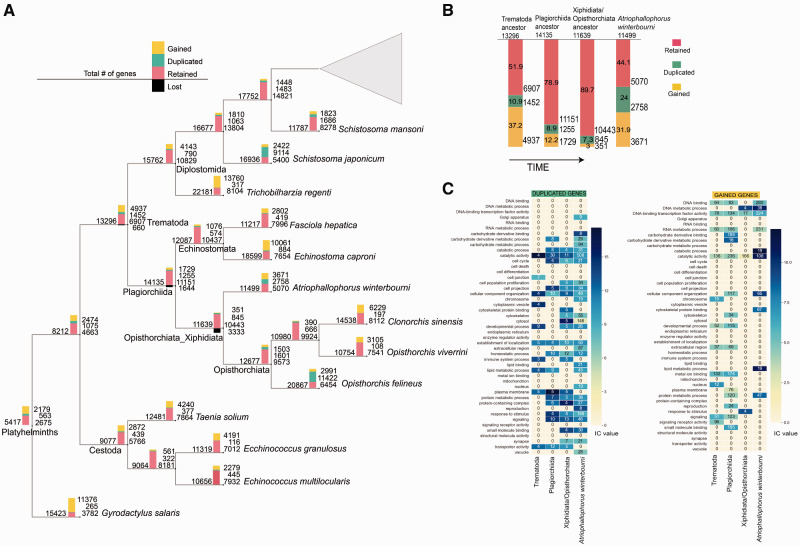
(*A*). Number of duplicated, retained (1:1 orthologs) and gained genes resulting after each point of speciation obtained from the analysis of HOG in pyHam, mapped onto a phylogenetic tree of trematodes (for original, see supplementary fig. S6, Supplementary Material online). The total number of genes at each point is indicated on the left-hand side of the bar and the total number of retained (pink), duplicated (green), and gained (yellow) genes are indicated on the right-hand side of the bar. The bars indicate the proportions of genes in each category. The lost genes are indicated only for the three ancestral genomes: the Trematoda ancestor, the Plagiorchiia ancestor, and the Opisthorchiata/Xiphidiata ancestor. (*B*) The proportions (on the bars) and the total numbers (next to the bars) of retained (pink), duplicated (green), and gained (yellow) genes in each reconstructed ancestral genome leading to *A. winterbourni*. The oldest ancestral genome is on the left-hand side and the extant *A. winterbourni* genome on the right-hand side. The total number of genes per genome is above each bar beneath the name. (*C*) Heatmaps summarising the GO enrichment analysis of the duplicated and gained genes in the three reconstructed ancestral genomes and the extant genome of *A. winterbourni.* All enriched GO terms were categorized into GO slims, listed on the *y*-axis of each heatmap. The colors indicate the mean IC value of each GO slim category and the number printed on top is the number of unique genes within that GO slim category (see Materials and Methods).

The OMA analysis identified 38,144 HOGs among all the species included (2 Nematodes and 18 Platyhelminthes). Specifically, in *A*. *winterbourni* 5,815 gene families were found (comprising 7,828 out of a total of 11,499 genes, 68.1%) with the rest being identified by OMA as singletons not belonging to any family (3,671 genes, 31.9%). Comparisons of three ancestral genomes among the trematode phylogeny (the ancestral Trematoda, the ancestral Plagiorchiida, and the Opisthorchiata/Xiphidiata ancestor) revealed many duplicated and gained gene families (fig. 3, supplementary fig. S7, Supplementary Material online). A particularly high proportion of genomic novelty was inferred during the initial speciation of Trematoda from the Trematoda/Cestoda common ancestor (37.2% of newly acquired genes), and again during the divergence of *A. winterbourni* from the most recent Opisthorchiata/Xiphidiata ancestor (31.9% of newly acquired genes, fig. 3*B*). The proportion of duplicated genes in the *A. winterbourni* genome was also high (24%) when compared with the Trematoda/Cestoda split (10.9%) (fig. 3*B*). In *A. winterbourni*, many of the duplicated genes were found in expanded gene families (503 genes comprising 66 HOGs with a minimum of 5 duplications per HOG) and 13 of these HOGs were massively expanded, with over ten duplicated genes since the Opisthorchiata/Xiphidiata speciation (supplementary table S4, Supplementary Material online).

We found only 660 genes lost in the ancestral Trematode from the previous ancestor. We observed a progressive increase in the number of lost genes to the Opisthorchiata/Xiphidiata ancestor (fig. 3*A*). The Plagiorchiida ancestor exhibited comparable gene loss to gene gain and duplication whereas in the Opisthorchiata/Xiphidiata ancestor, gene loss exceeded the number of duplications or gains (fig. 3*A*).

### 1:1 Orthologs in Trematodes

Based on previous studies, we assumed that many of the genes that remain conserved throughout speciation are housekeeping genes, the building blocks of the organism, and necessary for life, growth, and reproduction (Wu et al. 2006; Duarte et al. 2010). The prediction was confirmed through the GO annotations associated with the genes retained at a 1:1 orthologous gene ratio for each of the ancestral genomes (supplementary table S5, Supplementary Material online). The enriched GO terms for retained genes over all ancestors and *A. winterbourni* can be summarized as: RNA processing, the establishment of protein localization, organelle organization, embryo development, cellular catabolism, developmental process, reproduction, and response to stress and stimulus. What is more, since the ancestral trematode species 400 Ma, the number of genes retained at a 1:1 ratio remained relatively constant for each of the 14 extant trematodes, between 2,966 and 5,203 genes (supplementary table S6, Supplementary Material online).

Additionally, we found 28 single-copy orthologs present in all species, which have been maintained since the trematode ancestor. Examination of their functions through the annotations of best studied trematodes (*Fasciola hepatica* [NCBI 2017], *Schistosoma mansoni* [Protasio et al. 2012; Wang et al. 2016]) revealed that the 28 retained gene families shared between them all were largely involved in cell functioning and growth, division, and cell-to-cell or protein-to-protein interactions (supplementary results S4, table S7, Supplementary Material online).

### Genes Duplicated and Gained in Trematodes

We hypothesized that the duplicated genes are more likely to be adaptive than the single-copy orthologs due to the redundant second copy being functionally maintained through positive selection to play a new or same role within the organism (Ohno 2013; Yang et al. 2015). Multiple duplications within gene families would further suggest an adaptive importance of these key HOGs. The novel (gained) genes may similarly indicate areas of genetic innovation that were crucial in adoption of new hosts, expansion/streamlining of life cycles, and adaptation to changing environments. The origins of the gained genes may stem from neofunctionalization or high divergence of duplicated genes, therefore also potentially involved in adaptive functions as suggested by the gene duplication model of Ohno (2013).

Examination of the enriched functions from the trematode ancestor to the most recent ancestor of *A. winterbourni* are presented in figure 3*C* and appear to indicate a progressive gain and duplication of potentially adaptive genes. An “ancestral GO enrichment” analysis of the ancestral genomes was used to retrieve the putative functions of all gained genes (shared between at least 70% of the extant species in Trematoda, 66.7% of Plagiorchiida, or 50% of Xiphidiata/Opisthorchiata) and duplicated genes (minimum five duplicated genes per family) (supplementary methods S6, Supplementary Material online). Here, we concentrate on functional analysis of ancestral genomes because the inferred gene duplications, gains, and losses are based on evidence present in all of the extant genomes. For example, a gene is inferred to be gained at a particular ancestral level if it is present in at least two species only in that clade. Therefore, ancestral genomes (i.e., internal nodes in the species tree) are more robust than extant genomes in terms of inferred evolutionary duplications, gains, and losses. Additionally, by only considering gained genes present in the majority of the extant species of a given clade, or duplicated genes present with at least five copies, we have more confidence that we are looking at *bona fide* gains and duplications. The ancestral genome annotation was based on combining the GO terms assigned to the extant genomes. We further categorized the enriched GO terms into GO slim categories to give a broader overview of the functions and counted unique genes within each of those categories (summarized in fig. 3*C*). Although there was a similar number of enriched functions for the duplicated and gained genes in the Trematoda and Plagiorchiida ancestors, we found more functions enriched in duplicated than in gained genes in the Xiphidiata/Opisthorchiata ancestor and in *A. winterbourni*. Considering only the duplicated genes, from the trematode ancestral genome to the *A. winterbourni* genome, there was a progressive increase in the number of enriched GO slim functions over time, and an overall increase in the number of unique genes contributing to each function. The increase in the number of unique genes could possibly reflect the increasing importance of this function over time or increased duplication rate of certain families.

We present the average IC per GO slim category, which can be used as a proxy to estimate the specificity of a particular GO term (see Materials and Methods). The higher the IC, the more specific a term. For the gained genes, we found a progressive increase in IC value of the different GO slim categories but we did not find an increase in the number of enriched GO slim functions or the number of unique genes within them (fig. 3*C*). The increase in average IC values of GO slim categories enriched for gained genes could suggest an increase in specificity of functions over time (fig. 3*C*). These observations are best illustrated with enriched GO slim functions such as catalytic activity (GO:0003824), including microtubule motor activity, but also cellular component organization (GO:0005634), including actin bundle filament organization and response to stimulus. A literature review relates them to the importance of the microtubule-based and actin-based cytoskeletal system building the outer body layering (tegument), through which the parasite interacts with the host environment. Microtubule associated proteins in the tegument, including tubulin, paramyosin, actin, dynein light chains, and various antiporters, participate in absorption and secretion (e.g., nitrogen utilization), transport of vesicles from sub-tegumental cells to the tegument cytoplasm, and cell motility (Githui et al. 2009; Young et al. 2010). Molecular characterization and immunostaining studies have also shown dynein light chains to function as tegument associated antigens (Hoffmann and Strand 1997; Yang et al. 1999; Jones et al. 2004), important in hiding from host immunity. The tegument has been shown to be an essential structure for adaptation to the external environment (Kim et al. 2012) including the pH of the digestive system of the hosts. Indeed, our results show dynein light chain, tegument-associated antigen, and a tubulin-beta chain to be the functions of 3 of the 12 HOGs duplicated since the Trematode ancestor and with at least 3 copies in 75% of the extant species (supplementary table S8, Supplementary Material online). We also found dynein light chain to be the putative function of one of the most duplicated HOGs in *A. winterbourni* (supplementary table S4, Supplementary Material online, see next section), as well as a HOG duplicated in all 14 trematode species (supplementary table S9, Supplementary Material online). Thus, we speculate the functions related to the tegument to be also of great importance in our focal species.

The results might indicate acquisition of more complex and specific adaptations to hosts and environments over time. More experimentally validated GO annotations in our species of interest could shed light on this hypothesis in the future.

### Gene Loss in Trematodes

Gene loss is known to be common for intracellular parasites (Sakharkar et al. 2004; Corradi 2015) and it is much rarer in parasites with complex life cycles and multiple hosts (Zarowiecki and Berriman 2015). However, in several helminths there has been a loss of a mitochondrial gene *atp8* (Egger et al. 2017) or cytochrome P450 redox enzymes (Tsai et al. 2013) as well as other functional losses and gene family contractions (International Helminth Genomes Consortium 2019). Here, we again focused on ancestral genomes because they are inferred by the accumulation of gene presence and absence information from the extant genomes, that is, if a gene is not found in all the extant genomes of a clade, we can assume it was lost in the last common ancestor of that clade. Thus, ancestral genome analysis is less prone to being undermined by poorer quality genomes (Deutekom et al. 2019). In our study, the robustness was exhibited by the number of losses being always much lower in ancestral than extant genomes (supplementary fig. S7, Supplementary Material online). We also performed a GO enrichment of the lost genes for *A. winterbourni* as well as the ancestors leading to it. For the ancestral genomes, the background population for GO enrichment was the union of all the GO terms in the extant children species constituting the previous ancestor to the ancestor of interest.

Although there was a progressive increase in the number of genes lost from Trematoda to Opisthorchiata/Xiphidiata ancestor, a GO enrichment analysis of lost genes did not reveal any functions to be enriched in the Trematoda or Opisthorchiata/Xiphidiata ancestor. In the Plagiorchiida ancestor we found loss of genes related to intrinsic components of membrane (GO:0016021) and wide pore channel activity (GO:0022829). We did not find any enrichment of GO terms for the lost genes in *A. winterbourni*. Since the functions of the lost genes appear to not be related to any specific biological processes, we speculate that there is a greater importance of gene gains and duplications in adaptation to parasitism.

### Role of Gene Duplication and Gain in Driving Adaptation of *A. winterbourni*

The Opisthorchiata species exhibit a high similarity in life cycle traits and set of hosts. The *A. winterbourni* genome exhibited comparable proportions of gained, retained, and duplicated genes since the Opisthorchiata/Xiphidiata ancestor (31.9%, 44.1%, 24%, respectively) as *Opisthorchis viverrini* (41%, 50.5%, 8.5%, respectively), that is, in both species the highest proportion of genes was retained and the smallest proportion of genes was duplicated. On the other hand, *Opisthorchis felineus* exhibited a much higher proportion of genes originating through duplication since Opisthorchiata/Xipihidiata ancestor (52.4%) and *Clonorchis sinensis* had the most genes originating through gain since the Opisthorchiata/Xiphidiata ancestor (54.3%). Thus, across the four species, sometimes gene duplication and sometimes gene gain seems to play a greater role in gene family evolution. However, it is important to note that inferences regarding gene duplications, gains, and losses in extant species rather than ancestral species are impacted to a greater extent by fragmentation in genome assemblies, likely inflating the numbers in these categories of genes.

The *A. winterbourni* genome revealed a massive expansion of 13 HOGs that occurred after the speciation from Opisthorchiata/Xiphidiata ancestor (over ten duplicated genes/HOG, comprising 221 genes, supplementary table S4, Supplementary Material online). Comparing *A. winterbourni* to the Opisthorchiata/Xiphidiata ancestor, two gene families stood out due to the presence of more than 30 *A. winterbourni* genes: HOG 25969, with 31 genes in *A. winterbourni* out of 56 genes in all trematodes, and HOG 36190, with 36 genes in *A. winterbourni* out of 72 genes. In these two families, 29 and 31 genes originated through duplication since the Opisthorchiata/Xiphidiata ancestor for HOG 25969 and HOG 36190, respectively. In any other trematode, only 1–5 copies were found. These genes were investigated for being artificially duplicated due to a high proportion of BUSCO duplicated genes found within the assembly. Genes could be considered artificial duplications due to being fragmented by breaks between scaffolds (Alkan et al. 2011). We looked at the positions of the duplicated genes of HOG 25,969 and 36,190 on their scaffolds, and we did not find this to be the case (supplementary table S10, Supplementary Material online). We thus concluded our genes are likely real duplications rather than artificial duplications due to assembly fragmentation.

### Functions of Massively Expanded Gene Families in *A. winterbourni*

Examination of GO annotations of the 13 HOGs with over ten recently duplicated genes (supplementary table S4, Supplementary Material online) led us to speculate that the genes are likely involved in host tissue invasion and exploitation (metallohydrolases, Baskaran et al. 2017), escape from host immunity (serpins, Bao et al. 2018), and host behavioral manipulation (glutamine synthase, Helluy and Thomas 2010) (supplementary table S4, Supplementary Material online).

Specifically, we examined the two most highly duplicated gene families in depth. We determined HOG 36190 (36 genes in *A. winterbourni*) to be a gene family of putative glutamine synthases (supplementary table S4, Supplementary Material online). Already from the Plagiorchiida ancestor to the Opisthorchiata/Xiphidiata ancestor there was a significant enrichment in biological processes and cellular components related to glutamine family amino acid metabolic processes, including glutamate ammonia ligase activity (GO:0004356), positive regulation of synaptic transmission, glutamatergic (GO:0051968), glutamate binding (GO:0016595), glutamate catabolic process (GO:0006538), and glial cell projection (GO:0097386) (supplementary table S5, Supplementary Material online). The glutamine biosynthesis pathway is a pathway in which one of the end products is proline, a nonessential amino acid. An extremely active proline pathway has already been observed in most helminths infecting humans (*Fasciola hepatica*, Schistosomes), with host derived arginine used as a substrate (Ertel and Isseroff 1976; Toledo and Fried 2010; Mehlhorn 2016). These excessive proline levels have been implicated in the pathogenesis of trematode infections. Proline alters antioxidant defenses, activating secondary metabolite virulence factors, but also provides an energy source for a metabolic shift appropriate for adaptation to the host environment (Ertel and Isseroff 1976; Toledo and Fried 2010; Mehlhorn 2016). Glutamine synthase has also been found to be a marker for glial cells, immunity cells of the central nervous system. A study on *Microphallus papillorobustus*, a trematode parasite of *Gammarus* crustaceans, found disruption of the glutamine metabolism in the brain of the gammarids due to astrocyte-like glia and nitric oxide production by the parasite metacercariae, resulting in altered neuromodulation and behavior of the host (Helluy and Thomas 2010). The gene family is thus especially interesting and a potential candidate in parasite–host interactions as previous research has shown *A. winterbourni* to be affecting the behavior of its snail host (Feijen et al., in preparation; Levri and Lively 1996).

The second highest-duplicated gene family in *A. winterbourni* was HOG 25969, with 31 genes. It consists of proteins putatively encoding for O-sialoglycoprotein endopeptidase, tRNA N6-adenosine threonylcarbamoyltransferase, metallohydrolase, and/or glycoprotease/Kae1, all related to DNA repair, protein binding, and metal ion binding (supplementary table S4, Supplementary Material online). The GO annotation indicates the family to be potentially involved in DNA repair, nuclease activity, and nucleic acid phosphodiester bond hydrolysis. Metalloproteases have been found to be duplicated and under positive selection in other parasitic worms (*Strongyloides papillosus*), showing them to be involved in host tissue penetration at final larval stage (Baskaran et al. 2017).

### Signatures of Selection in Two Expanded Gene Families of *A. winterbourni*

**Fig. 4:**
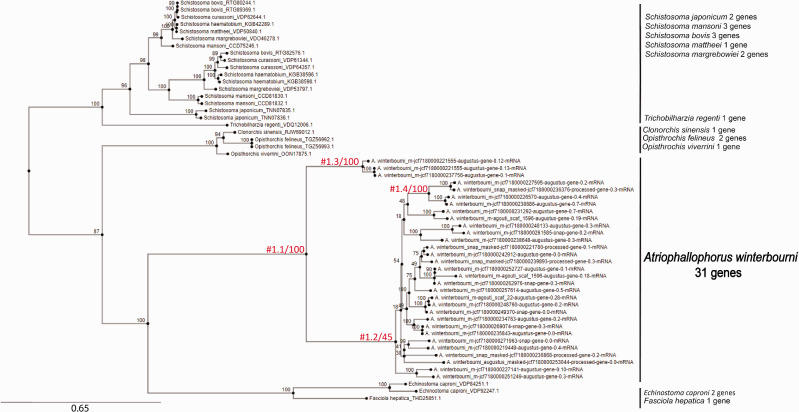
Gene tree of gene family HOG 25969 created with IQ-TREE. The tree is unrooted. Each name is a species name followed by the original gene name (protein name). *Atriophallophorus winterbourni* gene names are shortened version of gene names in supplementary table S10, Supplementary Material online. The numbers above branches indicate ultrafast bootstrap support, for the #1 branches the bootstrap support is after a backslash. The branches labelled with #1.X indicate the separation between the foreground branches and the background branches (distinction used in codeml for investigation of selection). The test for selection compares the d*N*/d*S* between the foreground branch and the background branches. The total number of genes in this HOG per trematode species is given next to each species name.

We next investigated the two highly duplicated (>30 genes) HOGs described above for signatures of positive selection. Signatures of positive selection were detected by comparing the d*N*/d*S* ratio at branches leading to the radiation of *A. winterbourni* genes, indicated with a #1 on the gene tree, with the dN/dS ratio of background branches (fig. 4, supplementary fig. S9, Supplementary Material online). Selection is generally considered negative/purifying if *ω* (or d*N*/d*S*) is less than 1, neutral if *ω* is 1, and positive if *ω* is greater than 1.

HOG 36190 was the most massively expanded HOG and selection was found to be acting on some but not all genes within this family. In the d*N*/d*S* ratio analysis, the null model (allowing *ω* ≤ 1) explained the data better than the alternative model (allowing *ω* > 1) for 2 out of 3 of the investigated branches, indicating neutral evolution (table 2, supplementary fig. S9, Supplementary Material online). The signature of selection was detected only on one branch, a long branch leading to a subset of 13 *A. winterbourni* genes within this family (supplementary fig. S9, Supplementary Material online, branch #1.3). Eleven sites were identified as >50% probability to be under positive selection with one having a probability >90%. From this we conclude that selection might be acting on some, but not all, genes within this family potentially indicating a certain structure evolving under positive selection. However, considering we do not find selection on any other branches in the gene tree, it also has to be taken into account that genes in this family might be highly proliferating due to being in genomic locations prone to duplication events. Their increasing number can be causing redundancy, which can ultimately be deleterious to the organism (Schiffer et al. 2016).

**Table 2:** Results of Studying Positive Selection in Two Majorly Expanded Gene Families in *Atriophallophorus winterbourni*

HOG	Node	LRT	df	*P*-value	Positively Selected Sites (Position in the Alignment, Amino Acid, Probability of Being under Positive Selection)			
36190	#1.1	0.00017	1	0.98	—			
36190	#1.2	1.9	1	0.17	—			
36190	#1.3	16.9	1	3.9E-05	291 A 0.725	874 L 0.731	1030-0.681	
					367 E 0.910	875 S 0.800		
					370 K 0.745	878 Y 0.564		
					371 K 0.827	879 V 0.518		
					873 K 0.626	880 P 0.707		
25969	#1.1	25.6	1	4.13E-07	603 A 0.767	794 A 0.575	922 I 0.548	998 V 0.504
					607 S 0.664	795 K 0.694	932 N 0.516	1038 Q 0.542
					638 N 0.707	798 I 0.662	951 K 0.683	1073 S 0.605
					649 V 0.935	803 S 0.762	955 H 0.878	1090 S 0.875
					675 S 0.669	804 G 0.556	970 T 0.513	1095 Y 0.951*
					680 C 0.912	812 R 0.893	976 Q 0.846	1121 S 0.508
					701 I 0.684	833 S 0.514	986 N 0.906	1184 R 0.931
					705 K 0.541	871 A 0.695	989 F 0.544	1188 I 0.521
					716 Y 0.852	879 Q 0.889	994 S 0.624	1198 H 0.700
					719 C 0.966*	921 N 0.983*	996 F 0.957*	
25969	#1.2	5.5	1	1.80E-02	366 K 0.537			
					394 W 0.534			
					464 K 0.661			
					473 N 0.832			
					637 T 0.623			
					657 H 0.830			
					662 D 0.692			
					665 S 0.707			
25969	#1.3	4.3	1	3.70E-02	402 R 0.867			
					873 F 0.624			
25969	#1.4	26.7	1	2.30E-07	370 T 0.756	831 N 0.534	1004 N 0.974*	
					394 W 0.559	857 E 0.984*	1029 F 0.676	
					480 R 0.868	928 S 0.766	1044 D 0.846	
					482 L 0.590	929 G 0.986*	1080 E 0.767	
					611 C 0.632	931 N 0.687	1157 N 0.674	
					710 N 0.697	932 N 0.546	1162 Y 0.635	
					714 S 0.733	967 H 0.955*	1184 R 0.922	
					722 M 0.740	970 T 0.811	1189 L 0.886	
					749 P 0.791	988 M 0.940		
					814 E 0.637	994 S 0.729		

Note.—The HOG indicates the ID of the gene family. The node relates to the nodes indicated in the gene trees of each HOG. LRT—results of likelihood ratio test, *P*-value is the result of chi^2^ test of the LRT. Positively selected sites are the result of BEB (Bayes empirical Bayes) test implemented in codeml. The starred values indicate sites under significantly high probability of selection (>95%).

For the gene family HOG 25969 the alternative model (allowing *ω* > 1) explained the data better than the null model (allowing *ω* ≤ 1) for all the investigated branches, indicating a signature of positive selection on all of the investigated foreground branches (table 2, fig. 4). With this result we followed up with the post-hoc Bayes Empirical Bayes (BEB) analysis implemented in the alternative model (Yang et al. 2005). For the branch leading to all 31 *A. winterbourni* genes, the BEB analysis identified 39 amino acids residues to be under positive selection in the alignment with 4 sites having an over 95% probability of being selected. For sites under positive selection among different subsets of foreground lineages, see table 3. Analysis of positive selection on the structures of the enzyme showed the active, DNA or mental binding site to be under highest probability of selection suggesting an important role (supplementary fig. S8, results S5, Supplementary Material online). However, without experimental characterization it is difficult to say what role the family might be playing in *A. winterbourni*.

## Conclusions

In our study, we report a de novo sequenced genome of a digenean trematode parasite, *A. winterbourni*, its phylogenetic position among other digenean trematodes, and the time of speciation of its ancestor from Opisthorchiata suborder. Using 14 other currently available and well-studied parasitic digenean trematodes, we reconstruct the ancestral trematode genome and investigate which genes have originated through duplication, which were gained and which have remained conserved (retained) through each speciation point until the extant genome of *A. winterbourni*. The comparative genomic approach is a powerful tool for identifying candidate duplicated gene families involved in adaptation. We find 13 gene families expanded recently in *A. winterbourni*, and for two we infer signatures of positive selection. Our description of candidate gene families putatively involved in parasite infectivity will facilitate the identification of genomic regions directly involved in the host–parasite coevolutionary arms race and will facilitate studying coadaptation in the laboratory. Gene expression studies in diverse life-cycle stages and functional confirmation via, for example, RNAi knock-out studies will be required to provide a direct link between the genes and phenotypes involved. By focusing on gene duplications and retention across the digenean trematodes our work informs on the genomic basis of adaptation to parasitic lifestyles and paves the way for future adaptation genomics focusing on antagonistic relationships between host and parasites.

## Supplementary Material

evab010_Supplementary_Data
