# Supplementary material for: Gene Duplication and Gain in the Trematode Atriophallophorus winterbourni Contributes to Adaptation to Parasitism
Source: Genome Biol Evol. 2021 Jan 23;13(3):evab010. doi: 10.1093/gbe/evab010 (PMC7936022; doi:10.1093/gbe/evab010)
Supplement: evab010_Supplementary_Data [file evab010_supplementary_data.zip › Supplementary Information for Comparative genomics of parasitic worms - GBE_revisions_clean.pdf]

Supplementary Information for:

Gene duplication and gain in *Atriophallophorus winterbourni* and other major parasitic trematodes contributes to adaptation to parasitism

Table of contents:

1. Supplementary methods
    a. **Supplementary methods 1**. Hatching adult worms from metacercariae for DNA extraction.
    b. **Supplementary methods 2.** Details of annotation of the protein coding sequence of the reference genome of *Atriophallophorus winterbourni*.
    c. **Supplementary methods 3.** The assessment of completeness and quality of the annotation
    d. **Supplementary methods 4.** Verification of experimental evidence for GO annotation of the *A. winterbourni* genome
    e. **Supplementary methods 5.** Filtering of codon alignments of HOGs for positive selection analysis.
    f. **Supplementary methods 6.** Code used for the analysis in jupyter notebooks:
        i. Each_ancestral_genome_reconstruction_until_Atriophallophorus
        ii. Extant_trematodes_compared_to_ancestral_trematode
        iii. Atriophallophorus_compared_to_Opisthorchiata_Xiphidiata_ancestor
    g. **Supplementary methods 7**. Study of protein structure of HOG 25969.
2. Supplementary Results
    a. **Supplementary results 1.** Genome assembly.

b. **Supplementary results 2.** Gene Ontology annotation of *Atriophallophorus winterbourni* using OMA, Pannzer2 and EggNOG.

c. **Supplementary results 3.** Quality assessment of annotation of protein coding sequences in the genome of *A.winterbourni* using full length transcript analysis.

d. **Supplementary results 4**. Protein length of duplicated, gained and retained genes in extant trematodes since the trematode ancestor.

e. **Supplementary results 5**. Study of protein structure of HOG 25969.

3. Supplementary Figures

a. **Supplementary Box 1**. Details of life cycles of digenean trematodes used in the study.

b. **Supplementary Figure 1.** Propidium Iodine staining plot showing peaks for *Drosophila melanogaster* (standard, haploid genome size 175 Mb) and *Atriophallophorus winterbourni*. PI stains all DNA therefore the genome size can be calculated from a relative measure between a standard and a focal sample.

c. **Supplementary Figure 2**. GenomeScope k-mer profile plot (21-mer frequency distribution) for Illumina filtered data. The first peak located at coverage 21X corresponds to the heterozygous peak. The second peak at coverage 42X, corresponds to the homozygous peak. Estimate of the heterozygous portion is 3.6%.

d. **Supplementary Figure 3**. BlobPlot of the *A.winterbourni* assembly. The assembly was assessed for taxonomic uniformity using Blobtools 0.9.19.5. All assembly scaffolds are depicted as circles and the diameter of the circle indicates sequence length. All scaffolds >50 000bp and a random sample of scaffolds <50 000bp from assembly were submitted to BLAST using NCBI nr database for taxonomic annotation. The circles ae coloured by taxonomic annotation. Grey indicates the scaffold was not submitted to BLAST. Circles are

positioned according to their proportion of GC content and the coverage of their reads which map onto the scaffolds.

e. **Supplementary Figure 4**. Frequency distribution of the total length of proteins of 14 species of trematodes used in the analysis. The higher peaks belong to genomes with BUSCO completeness score (proportion of single copy orthologs) below 45% indicated by an arrow on the figure and by a star on the legend) and the bottom belong to genomes with BUSCO completeness score above 45%. *Atriophallophorus winterbourni* falls into the group of genomes with higher BUSCO completeness and greater number of longer proteins.

f. **Supplementary Figure 5**. The number of functionally annotated genes of *Atriophallophorus winterbourni* by Pannzer2, OMA, EggNOG and their overlap in the genes they annotated. All three programs annotated 1281 genes.

g. **Supplementary Figure 6**. Species tree created with ASTRAL III. 238 gene trees for 238 Orthologous Groups were created with MAFFT and together used in ASTRAL III to create a unifying species tree.

h. **Supplementary Figure 7**. Original tree created in pyham showing total number of gained, duplicated, retained and lost genes on each branch of the phylogeny of species used in this study.

i. **Supplementary Figure 8**. **A**. A HOG 25969 PBD model (red) superimposed with DNA bound FEN1 nuclease (5V07, white) viewed from two different rotations. Green colour indicates sites with more than 90% probability of being under selection. Phe280 and Tyr127 indicate two sites are within two alpha helices which correspond to part of the typical DNA binding site present in FEN1 nucleases and show a positive selection at the site of two aromatic residues. The site numbers correspond to the MSA alignment on which the model was based

and are equivalent to sites 996 (Phe280) and 1095 (Tyr127) in Table 4 for branch #1.1 **B**. A zoom-in image onto the sites under selection.

j. **Supplementary Figure 9.** Gene Tree of gene family HOG 36190 created with IQ-TREE. The tree is unrooted. Each original gene name (protein name) is followed by the species name, the names of genes of *A. winterbourni* are referring to the names in Supplementary Table 11. The numbers above branches indicate bootstrap support, for the #1 branch the bootstrap support is separated with a slash. The #1 indicated the separation between the foreground branches and the background branches (distinction used in codeml for investigation of selection). The test for selection compares the dN/dS between the foreground and the background branches.

Supplementary Methods

1. Hatching adult worms from metacercariae for DNA extraction.

Metacercariae were obtained from each snail separately through dissection under the microscope. To initiate hatching, the metacercariae were incubated at 40 °C for 2-4 h in Tyrode's salt solution, supplemented with pancreatin (Sigma P3292) (0.15g/50ml of Tyrode's salt solution), 100 mg/mL Penicilin G (Fluka 13752) and 0.1g/mL of Streptomycin (Fluka 85880). For Tyrode's salt solution we mixed Tyrode's salts (Sigma T2145-10x1L) with 1L of MiliQ water and 1g of sodium bicarbonate ($NaHCO_3$). After all the worms had hatched to their adult stage they were washed twice with Tyrode's salt solution and antibiotics (100 mg/mL Penicillin G, Fluka 13752 and 0.1g/mL Streptomycin, Fluka 85880) to remove all the remaining cysts shed by the hatched worms, and transferred to a 1.5 mL tube (Eppendorf, safe lock). The worms were immersed in no more than 10µl of washing solution and washed with 200 µl of PBS to be immediately used for DNA extraction.

2. Details of annotation of the protein coding sequence of the reference genome of *Atriophallophorus winterbourni.*

Coding sequence annotation was performed by Maker 2.31.9. Transcriptome data (Bankers & Neiman, 2017) from the same species, available at DDBJ/EMBL/GenBank under the accession GFFK00000000, was used as input together with the invertebrate protein database from UniProt for initial creation of gene models that were then subsequently used to train gene finding tools Augustus 3.2.1 and SNAP version 2006-07-28. Transcriptomic evidence from our species as well as closely related species (*Schistosoma mansoni* (Protasio et al. 2012) and *Caenorhabditis elegans* (Kaletsky et al. 2018)) provided a more accurate result for the gene models. We produced a customized species library of repetitive elements for the annotation using RepeatModeler 1.0.11

and RepeatMasker 4.0.7 on the scaffolds (Smit et al., 2015; Smit & Hubley, 2018). All repetitive content collected by RepeatModeler was then submitted to blastx 2.3.0 to confirm that no proteins, hypothetical proteins or coding sequences were excluded from annotation. The confirmed repetitive content has been masked from all rounds of Maker annotation.

3. Assessment of completeness and quality of the annotation

The completeness and quality of the annotation was assessed with BUSCO and with full-length protein coding sequence analysis using BLAST+ according to the following protocol: https://github.com/trinityrnaseq/trinityrnaseq/wiki/Counting-Full-Length-Trinity-Transcripts (last accessed: 11.2018). The "Trinity transcript" analysis was done to assess how many genes are full length or nearly full length. All coding sequences from Maker annotation were compared to all proteins from the UniProt database (558681 proteins, realase 7.11.2018) using blastx (e-value 1e-20).

4. Verification of experimental evidence for GO annotation of the *A. winterbourni* genome

GO annotation of the *A. winterbourni* genome was performed with Pannzer2, OMA and EggNOG (see main methods). QuickGO was used to check all GO terms for having experimental evidence in manual assertion (ECO:0000269), in mutant phenotype evidence in manual assertion (ECO:0000315), in high throughput direct evidence in manual assertion (ECO:0007005) or in high throughput mutant phenotypic evidence used in manual assertion (ECO:0007001) in trematodes or nematodes (species: *Schistosoma haematobium*, *Schistosoma japonicum*, *Schistosoma mansoni*, *Schistosoma mattheei*, *Fasciola hepatica*, *Clonorchis sinensis* and *Caenorhabditis elegans*, taxon identifiers in https://www.uniprot.org/taxonomy/: 6185, 6182, 6183, 31246, 6912, 6183, 31246, 6912, 79923, 6239 respectively) (https://www.ebi.ac.uk/QuickGO/annotations (last accessed: 9.03.2020)).

5. Filtering of codon alignments of HOGs for positive selection analysis.

Positive selection analyses are sensitive to alignment errors. That is why we used a filtering approach suggested in Selectome database (Moretti et al., 2014) for HOG 36190 alignment ridden with gaps. Short, low quality sequences disrupting the alignment were filtered with MaxAlign leaving 55 sequences (out of 72). The alignment was performed with Clustal Omega and quality scores of each amino acid were computed with MCoffee 11.00.d625267 and Guidance2. Amino acids with quality scores below 93% in Guidance2 and 60% (below "Good") in MCoffee were replaced with "X" and the alignment was converted to codon alignment with PAL2NAL. Poor alignment columns were filtered out using TrimAl v1.3 (noallgaps, columns composed only of gaps).

6. See Jupyter notebook for supplementary method 6.

7. Study of structure of HOG25969.

To get a better description of this highly expanded gene family with no evident functional annotation or obvious homology to known proteins, we used remote homology detection and structural modelling tools to gain further insight. In order to model each of the sequences in the expanded family, we chose to use modeller (Webb and Sali 2016) in tandem with HHBlits to select appropriate structural templates, align them by taking into account structural constraints and finally generate models for each sequence by using multiple crystal structure templates. This approach allows for template based modelling using limited computational resources and enables modelling each member of the expanded family to allow for comparative analysis between models if needed. To generate a query alignment for HHBlits (Hildebrand et al., 2009; Remmert et al., 2011), the sequences assigned to HOG25969 from the *A. winterbourni* genome were aligned with Clustal Omega (Sievers & Higgins, 2014) for 3 iterations on default parameters. The resulting MSA was used to query the pdb70 database with HHBlits. Details on the content and construction

of the pdb70 database are available on the mmseqs (Mirdita et al., 2017) website(https://uniclust.mmseqs.com/, last accessed 04.2020). The protein structure was coupled with BEB results of the probability of selection on each base from the codeml analysis with #1.1 as the foreground branch (see methods Estimation of dN/dS in gene families in *Atriophallophorus winterbourni*). A pymol (PyMOL | pymol.org) plugin was written to color the residues of models with the p-values calculated for their positive selection to visualize positive selection in an intuitive way on the final models.

References:

Bankers, L., & Neiman, M. (2017). De novo Transcriptome Characterization of a Sterilizing Trematode Parasite (Microphallus sp.) from Two Species of New Zealand Snails. *G3: Genes|Genomes|Genetics*, *7*(3), 871.

Hildebrand, A., Remmert, M., Biegert, A., & Söding, J. (2009). Fast and accurate automatic structure prediction with HHpred. *Proteins*, *77 Suppl 9*, 128–132.

Kaletsky, R., Yao, V., Williams, A., Runnels, A. M., Tadych, A., Zhou, S., Troyanskaya, O. G., & Murphy, C. T. (2018). Transcriptome analysis of adult Caenorhabditis elegans cells reveals tissue-specific gene and isoform expression. *PLoS Genetics*, *14*(8), e1007559.

Mirdita, M., et al. (2017). Uniclust databases of clustered and deeply annotated protein sequences and alignments. *Nucleic Acids Research*, *45*(D1), D170–D176.

Moretti, S., et al. (2014). Selectome update: quality control and computational improvements to a database of positive selection. *Nucleic Acids Research*, *42*(Database issue), D917–D921.

Protasio, A. V., Tsai, I. J., et al. (2012). A systematically improved high quality genome and transcriptome of the human blood fluke Schistosoma mansoni. *PLoS Neglected Tropical Diseases*, *6*(1), e1455.

Remmert, M., Biegert, A., Hauser, A., & Söding, J. (2011). HHblits: lightning-fast iterative protein sequence searching by HMM-HMM alignment. *Nature Methods*, *9*(2), 173–175.

Sievers, F., & Higgins, D. G. (2014). Clustal Omega, accurate alignment of very large numbers of sequences. *Methods in Molecular Biology* , *1079*, 105–116.

Smit, A. F. A., & Hubley, R. (2018). *2008–2015. RepeatModeler Open-1.0*.

Smit, A. F. A., Hubley, R., & Green, P. (2015). *RepeatMasker Open-4.0. 2013–2015*.

Supplementary Results

1. Genome assembly.

The sequencing data was obtained using Illumina and Pacific Biosciences technologies (for number of reads at each stage, see Supplementary Table 3). After filtering, 244,185,272 paired Illumina reads and 1,981,809 PacBio reads spanning 1 to 35 kb in length were used for the assembly. Due to the high heterozygosity of the Illumina data (assessed by 21-mer frequency distributions, Supplementary Fig 2) and the inflation of the genome size in the initial assembly, we used Redundans for correction. We used the resulting assembly, combined with the existing *A. winterbourni* transcriptome (Bankers & Neiman, 2017), to submit to AGOUTI to improve the scaffolding and contiguity of the assembly (Zhang et al., 2016). The scaffolds ranged from 3,947 bp to 525,914 bp with an N50 of 40,108. Blobtools showed that 63.7% of the reads map to no taxonomic groups as those scaffolds have not been blasted (Supplementary Fig 3). Out of the 5739 scaffolds submitted to BLAST, 1069 scaffolds mapped to Platyhelminths (Supplementary Fig 3). 644 scaffolds mapped to Mollusca amounting to a total of 0.92% of the length of the 5739 scaffolds. Due to such negligible contamination with potential host DNA, Blobtools output was not used to filter the assembly. On the other hand, to eliminate any concern about host contamination, the *Potamopyrgus antipodarum* transcriptome obtained from (http://bioweb.biology.uiowa.edu/neiman/download.php, date accessed 15.07.2020) (Wilton et al. 2013) was used to filter the assembly. The host genome was converted to BLAST database and *A. winterbourni* genes were mapped to it searching for 100% matches (e-value = 1e-7). Consequently, 4 genes were eliminated. The assembly yielded 70.1% of the complete BUSCO genes, including 54.5% single copy and 15.6% duplicated genes.

2. Gene Ontology annotation of *Atriophallophorus winterbourni* using OMA, Pannzer2 and EggNOG.

OMA, Pannzer2 and EggNOG (Altenhoff et al., 2017; Huerta-Cepas et al., 2016; Törönen et al., 2018) were used for Gene Ontology annotation of the *A.winterbourni* genome. All three algorithms perform orthology-based GO annotation; however, each of them uses a different database of species and they differ in prioritizing precision vs coverage. We used all three to better understand our results. Pannzer2 annotated 5222 genes, yielding 88,789 gene-GO term combinations; OMA annotated 8106 genes, yielding 42,253 gene-GO term annotations; and EggNOG annotated 3032 genes, yielding 413,485 gene-GO term annotations (Supplementary Fig 5). From EBI QuickGO annotations we compiled a dataset of experimentally annotated GO terms from nematodes and trematodes (species: *Schistosoma haematobium, Schistosoma japonicum, Schistosoma mansoni, Schistosoma mattheei, Fasciola hepatica, Clonorchis sinensis* and *Caenorhabditis elegans*, see Methods for details). The dataset was 30,907 GO terms. We compared the GO terms annotated in *A.winterbourni* to this database. We found 28,255 GO terms out of 88,789 (32%) for the Pannzer2 GO annotation, 19,164 GO terms out of 42,253 (45.3%) for the OMA GO annotation and none of the EggNOG annotations to be based on experimental evidence in nematodes or trematodes (see methods).

3. Quality assessment of annotation of protein coding sequences in the genome of *A. winterbourni* using full length transcript analysis.

A "Trinity transcript" analysis using BLAST+ was performed on proteins annotated with Maker by comparing them to already existing sequences from other species (all proteins from the UniProt database, release 7.11.2018, 558681 proteins) to assess how many proteins are full length or nearly full length. It has to be taken into account that only a single best matching coding sequence is reported for each top matching database entry; in other words, if multiple proteins match a database entry or if any database entry matches multiple proteins from the studied species each protein is counted once along with a best matching database entry (highest BLAST score) and the rest of the results are discarded. Thus the results have to be understood as a measure of

improvement of annotation over multiple Maker annotation rounds rather than an absolute assessment of completeness of the assembly. The first round of Maker annotation yielded 1747 proteins covered with more than 80% of their protein length (3349 covered with more than 10% of their full length) and the final round of Maker annotation yielded 2317 proteins covered more than 80% of their protein length (4409 covered with more than 10% of their full length).

4. Comparison of extant trematodes to the ancestral trematode genome.

According to our analysis, the ancestral trematode had 13296 genes, most of which were retained since speciation from Cestoda (6907 genes, 51.9%), 1452 which originated through duplication (10.9%), and 4937 of which were newly acquired (37.2%) (Fig 3). Subsequently, we compared each extant trematode species to that of the ancestral genome to trace the evolutionary history of the extant genomes, focusing on duplicated and retained genes since the trematode common ancestor. We then used these categories of extant genes to find shared gene families between *A. winterbourni* and other trematodes.

The total number of 1:1 orthologous (retained) gene families per species can be seen in Supplementary Table 6. As mentioned in the main results there were 28 gene families which were retained at a 1:1 orthologous gene ratio in all 14 trematode species (Supplementary Table 7). Examination of their putative functions through the annotations of best studied trematodes (*Fasciola hepatica* (NCBI, 2017), *Schistosoma mansoni* (Protasio et al., 2012; Wang et al., 2016)) showed that the retained gene families were largely involved in cell functioning and growth, division and cell-to-cell or protein-to-protein interactions. However, Gene Ontology enrichment analysis of genes from every species from these HOGs showed no enrichment of any specific GO functions (Supplementary Table 12).

We studied duplicated gene families (or HOGs) with at least 3 genes that originated through duplication since the most recent common ancestor of all trematodes. The total number per species can be seen in Supplementary Table 6. There were 12 families with at least 3 duplicated genes, shared between at least 11 trematode species (Supplementary Table 8). For the putative functions of these families based on annotation of most well-known and studied trematode species (*Fasciola hepatica* (NCBI, 2017), *Schistosoma mansoni*,(Protasio et al., 2012; Wang et al., 2016)) see Supplementary Table 8 and for GO enrichment analysis results see Supplementary Table 12.

There were 2 duplicated gene families, HOG 33101 and HOG 35588, present in all 14 trematode species (138 and 105 genes respectively). The putative functions of these gene families based on BLASTP showed them to be dynein light chain type 1 (HOG 33101) and cathepsin B-like peptidase (HOG 35588) and GO annotation of genes from this family for all species confirmed the findings. GO enrichment analysis of the 138 genes from HOG 33101 indicated the genes to be implicated in dynein complex and microtubule based process. GO enrichment of 105 genes from HOG 35588 indicated the genes to be involved in cysteine-type endopeptidase activity and production of pigment granule (Supplementary Table 9).

5. Protein structure of HOG 25969.

One domain appears to be conserved across the entire HOG 25969, having an appropriate structural template which is detectable using HHBlits, FEN1 nuclease family of enzymes. Several sites of the domain show significant positive selection according to the codeml BEB results of the #1.1 branch (Fig 4). We were able to leverage our existing knowledge of the FEN1 structure (pbd70 database) and our observations relative to the selection of the nucleotide sequence of this gene family to gain possible insight into functionally important sites of the final protein. Multiple FEN1 nuclease templates mapped with ~15% identity to the template sequences. With a low

mapping identity, we could draw limited conclusions from Angstrom scale assessment of the models and thus we would like to draw attention to multiple other factors that could influence the folding or conformation of the FEN1 domain including other domains of the protein which are not modelled in our study and the DNA typically bound to FEN1 while optimizing the placement of side chains which typically results in more accurate modelling. The residues of the domain were coloured with the probability values of them being under positive selection (see Supplementary method 7) and two residues stood out due to having high (>90%) probability of being under positive selection and appear as highly conserved in the protein sequence alignment, Phe280 and Tyr127 (see Supplementary Fig. 9). The two sites are within two alpha helices which correspond to part of the typical DNA binding site present in FEN1 nucleases and show a positive selection at the site of two aromatic residues (Shi et al., 2017). This is particularly important for DNA binding enzymes since pi stacking interactions involving aromatic rings enable binding between the DNA and the enzyme by lowering the overall energy through a configuration where aromatic and conjugated double bonds in the DNA and enzyme are in close proximity. This pi-stacking interaction, which is dependent on the orientation and distance of the aromatic residue and nucleic acid, has a binding energy of approximately –43 kJmol−1 (Gallivan & Dougherty, 1999). That these sites would be positively selected in this expanded gene family may point to their importance in the DNA binding function of this particular gene product. However, no robust evolutionary explanations or conclusions can be drawn on the recent expansion without in-vivo experimentation.

References:

Altenhoff, A. M., et al. (2017). The OMA orthology database in 2018: retrieving evolutionary relationships among all domains of life through richer web and programmatic interfaces. Nucleic Acids Research, 46(D1), D477–D485.

Bankers, L., & Neiman, M. (2017). De novo Transcriptome Characterization of a Sterilizing Trematode Parasite (Microphallus sp.) from Two Species of New Zealand Snails. G3: Genes|Genomes|Genetics, 7(3), 871.

Campbell, M. A., et al. (2007). Identification and Characterization of Lineage-Specific Genes within the Poaceae. Plant Physiology, 145(4), 1311–1322.

Eisenberg, E., & Levanon, E. Y. (2003). Human housekeeping genes are compact. Trends in Genetics: TIG, 19(7), 362–365.

Gallivan, J. P., & Dougherty, D. A. (1999). Cation-π interactions in structural biology. Proceedings of the National. https://www.pnas.org/content/96/17/9459.short

Huerta-Cepas, J., et al. (2016). eggNOG 4.5: a hierarchical orthology framework with improved functional annotations for eukaryotic, prokaryotic and viral sequences. Nucleic Acids Research, 44(D1), D286–D293.

Luis Villanueva-Cañas, J., et al. (2017). New Genes and Functional Innovation in Mammals. Genome Biology and Evolution, 9(7), 1886–1900.

NCBI. (2017, November 9). Fasciola hepatica genome assembly, unpublished. NCBI F_hepatica_1.0.allpaths.pg. https://www.ncbi.nlm.nih.gov/assembly/GCA_002763495.1

Protasio, A. V., et al. (2012). A systematically improved high quality genome and transcriptome of the human blood fluke Schistosoma mansoni. PLoS Neglected Tropical Diseases, 6(1), e1455.

Shi, Y., Hellinga, H. W., & Beese, L. S. (2017). Interplay of catalysis, fidelity, threading, and processivity in the exo- and endonucleolytic reactions of human exonuclease I. Proceedings of the National Academy of Sciences of the United States of America, 114(23), 6010–6015.

Törönen, P., Medlar, A., & Holm, L. (2018). PANNZER2: a rapid functional annotation web server. Nucleic Acids Research, 46(W1), W84–W88.

Wang, T., Zhao, M., et al. (2016). Proteomic Analysis of the Schistosoma mansoni Miracidium. PloS One, 11(1), e0147247.

Wilton, P. R., Sloan, D. B., Logsdon, J. M., Jr, Doddapaneni, H., & Neiman, M. (2013). Characterization of transcriptomes from sexual and asexual lineages of a New Zealand snail (Potamopyrgus antipodarum). *Molecular Ecology Resources*, *13*(2), 289–294.

Zhang, S. V., Zhuo, L., & Hahn, M. W. (2016). AGOUTI: improving genome assembly and annotation using transcriptome data. GigaScience, 5(1), 31

Supplementary Figures

Life cycle of *Atriophallohporus winterbourni*

The final host of *Atriophallophorus* is waterfowl. The ducks feed on the aquatic snails; the trematode enters the digestive system of its final host as encysted larvae (metacercariae). The hermaphroditic worms hatch to their adult stage within the gut of the waterfowl. The worms cross-fertilize producing eggs that are released with the bird's faeces. Consequently, the eggs are released into the water where they are passively ingested by the snails feeding on algae on the rocks. When ingested by the snail, the egg hatches into a cercariae that moves into the reproductive tract of the snail and reproduces sexually into about 200-1000 metacercariae consequently castrating the snail.

Life cycle of *Schistosoma* species

Humans are often the final host but the species are rather generalist. The eggs are released from the definitive host into freshwater through feces or urine, and the eggs hatch in water releasing ciliated miracidia. The miracidia actively invade a snail host (*Biomphalaria*, *Bulinus* or *Oncomelania* depending on the species of *Schistosoma*). Inside the snail, the miracidia develop into cercaria and reproduce asexually. Attracted by light, the cercaria with forked tails leave the host during daylight hours. They penetrate the skin of the vertebrate host while leaving their tails and develop into schistosomulae. Eventually they gain access to the host's lymphatic system where (in different tissues depending on the species of *Schistosoma*) they develop into adults. The adult stages cause severe symptoms in their final hosts.

Life cycle of *Trichobilharzia regenti*

The life cycle of *Trichobilharzia regenti* is somewhat analogous to the one of *Schistosoma* species. The adult flukes mate in the nasal mucosa of water birds and produce eggs which hatch into miracidae. The miracidiae leak out of the tissue during feeding or eating. They swim to the intermediate host (snail species from genus *Radix*) where they develop into sporocysts and eventually cercariae. Cercariae are released and penetrate directly the skin of their avian host. They shed the glycoprotein layer and the tail and transform into schistosomula. The schistosomula moves through the nervous system into the brain and eventually into the nasal tissue in the bill.

Life cycle of *Fasciola hepatica*

The eggs are released with stool from cattle, sheep or buffalos into freshwater. Humans are also often the final host. The eggs hatch into miracidia, which find a suitable snail host (Lymnaidae family). In the snail, the miracidia develop into sporocysts, then rediae, then cercariae. The cercariae are released from the snail and form cysts on different surfaces including aquatic vegetation, which is then consumed by the mammalian hosts. The cercariae bury into the intestine walls, and gradually make their way into the bile duct where they develop into adult flukes.

Life cycle of *Echinostoma caproni*

Echinostoma caproni has a three-host life cycle. The two intermediate hosts belong either to the *Lymnaidae* family. In the first intermediate host, the miracidium undergoes asexual reproduction; the metacercariae transform into sporocysts, radiae and then cercariae. The free-swimming cercariae are released from the second host and penetrate the second intermediate host. The final host (usually an aquatic bird) becomes infected through consumption of the second intermediate host. Humans are often the final host.

Life cycle of *Clonorchis sinensis*

Clonorochis sinesis has a three-host life cycle. The definitive host are usually humans or other animals that consume raw freshwater fish. The metacercariae penetrate the intestine walls and move towards the bile duct. The eggs are released with feces and are ingested by the first intermediate host (most often a freshwater snail *Parafossarulus manchouricus*). The metacercariae hatch and develop into sporocysts, radiae and cercariae. The cercariae are not free swimming. When released the initially hang at the surface of water and then sink to the bottom. This movement is repeated several times until the cercariae feel the disturbance of water by fish which they attack. They burrow their way through the fish scales and develop in muscles.

Life cycle of *Opisthorchis* species

Opisthorchis viverrini (Southeast Asian liver fluke) and *Opisthorchis felineus* (cat liver fluke) have a very similar life cycle to the closely related *Clonorchis sinensis*. Human is the usual definite host for the two species (other hosts are those eating raw freshwater fish, including human pets). The eggs are released in feces into freshwater bodies. They are consumed by the first intermediate host, snails from the genus Bithynia. Within the snail the parasite undergoes all stages of development (miracidia, sporocysts, radiae and cercariae) and the free swimming cercariae are released. They attack freshwater fish, encysting in their muscles.

Information from: (Dybdahl and Lively 1996, Krist and Lively 1998, Esch, Barger et al. 2002, Galaktionov and Dobrovolskij 2003, Coon 2005, Hechinger 2012, Wanger, Chavez et al. 2017, (CDC) 2018)

Supplementary Box 1. Details of life cycles of digenean trematodes used in the study.

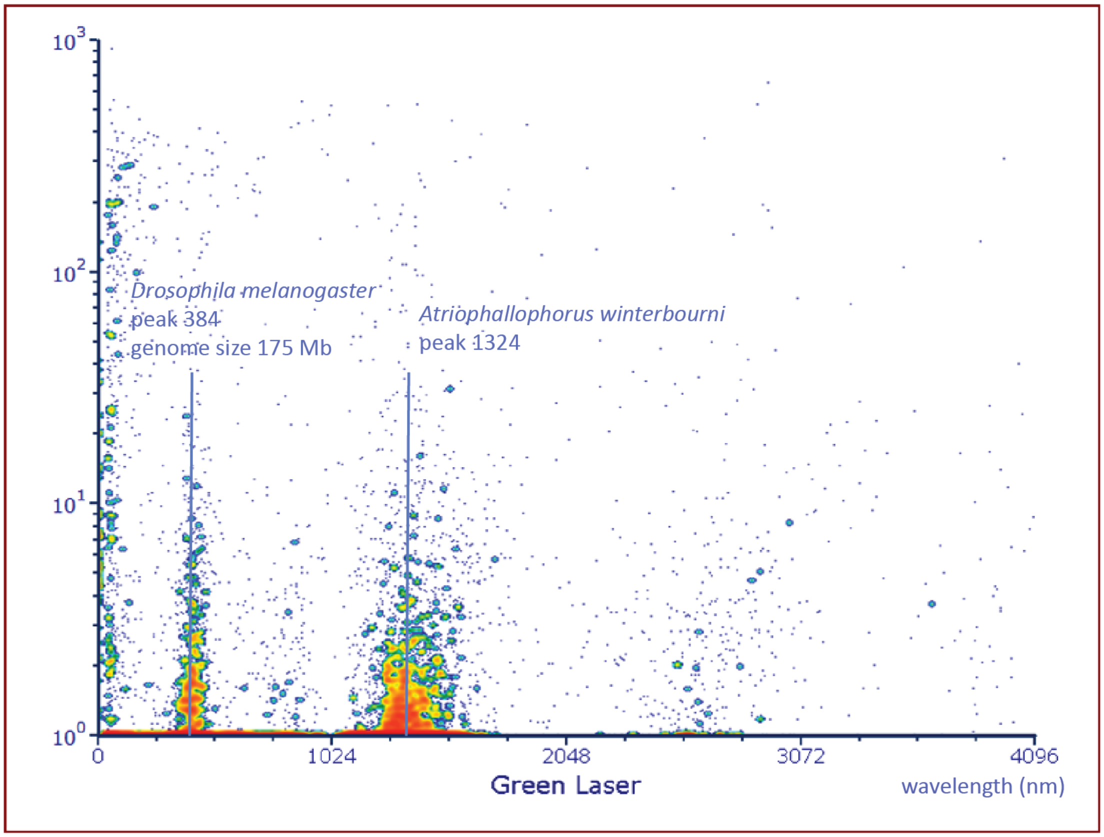

Supplementary Figure 1. Propidium Iodine staining plot showing peaks for *Drosophila melanogaster* (standard, haploid genome size 175 Mb) and *Atriophallophorus winterbourni*. PI stains all DNA therefore the genome size can be calculated from a relative measure between a standard and a focal sample.

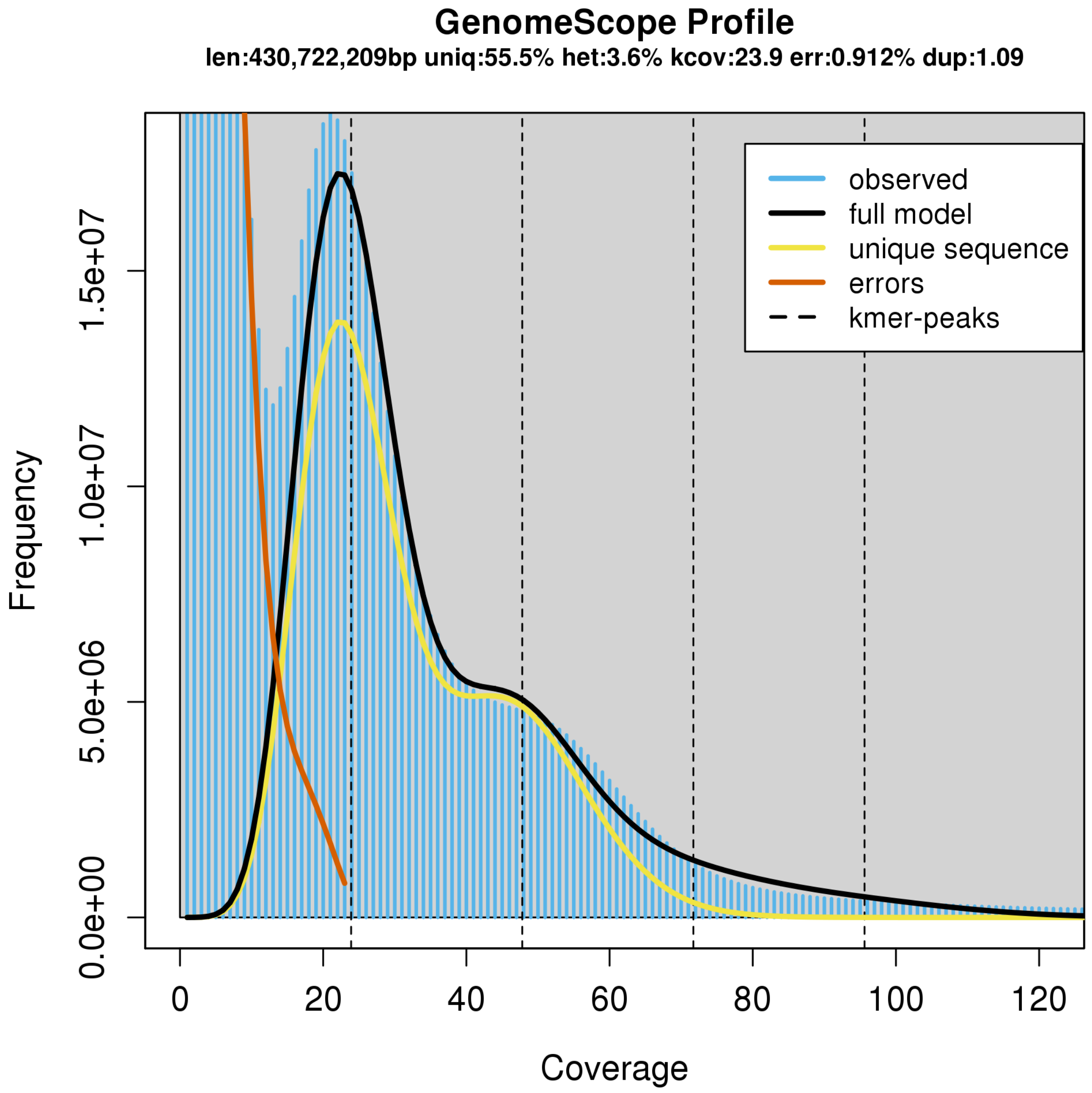

Supplementary Figure 2. GenomeScope k-mer profile plot (21-mer frequency distribution) for Illumina filtered data. The first peak located at coverage 21X corresponds to the heterozygous peak. The second peak at coverage 42X, corresponds to the homozygous peak. Estimate of the heterozygous portion is 3.6%.

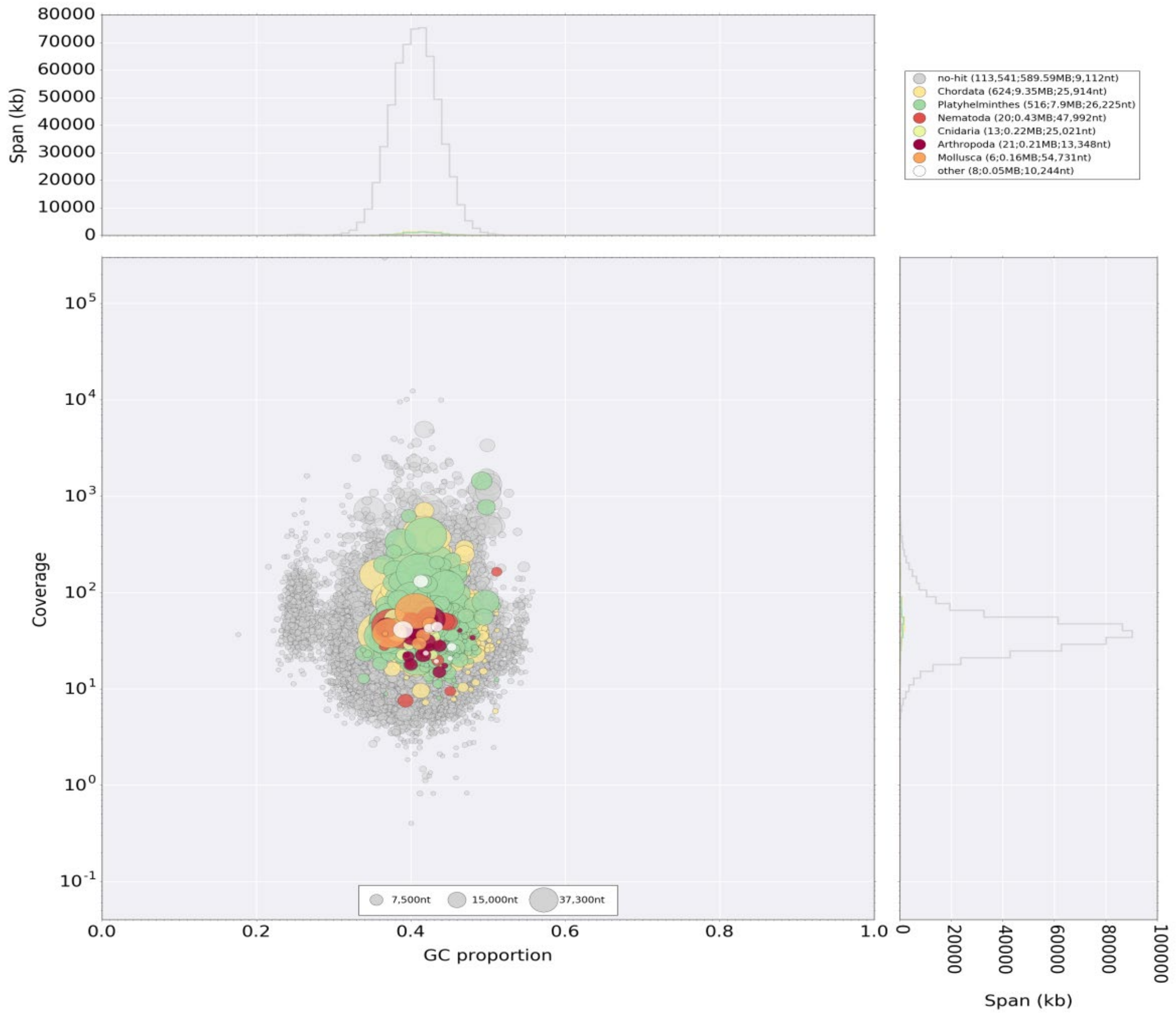

Supplementary Figure 3. BlobPlot of the *A.winterbourni* assembly. The assembly was assessed for taxonomic uniformity using Blobtools 0.9.19.5. All assembly scaffolds are depicted as circles and the diameter of the circle indicates sequence length. All scaffolds >50 000bp and a random sample of scaffolds <50 000bp from assembly were submitted to BLAST using NCBI nr database for taxonomic annotation. The circles ae coloured by taxonomic annotation. Grey indicates the scaffold was not submitted to BLAST. Circles are positioned according to their proportion of GC content and the coverage of their reads which map onto the scaffolds.

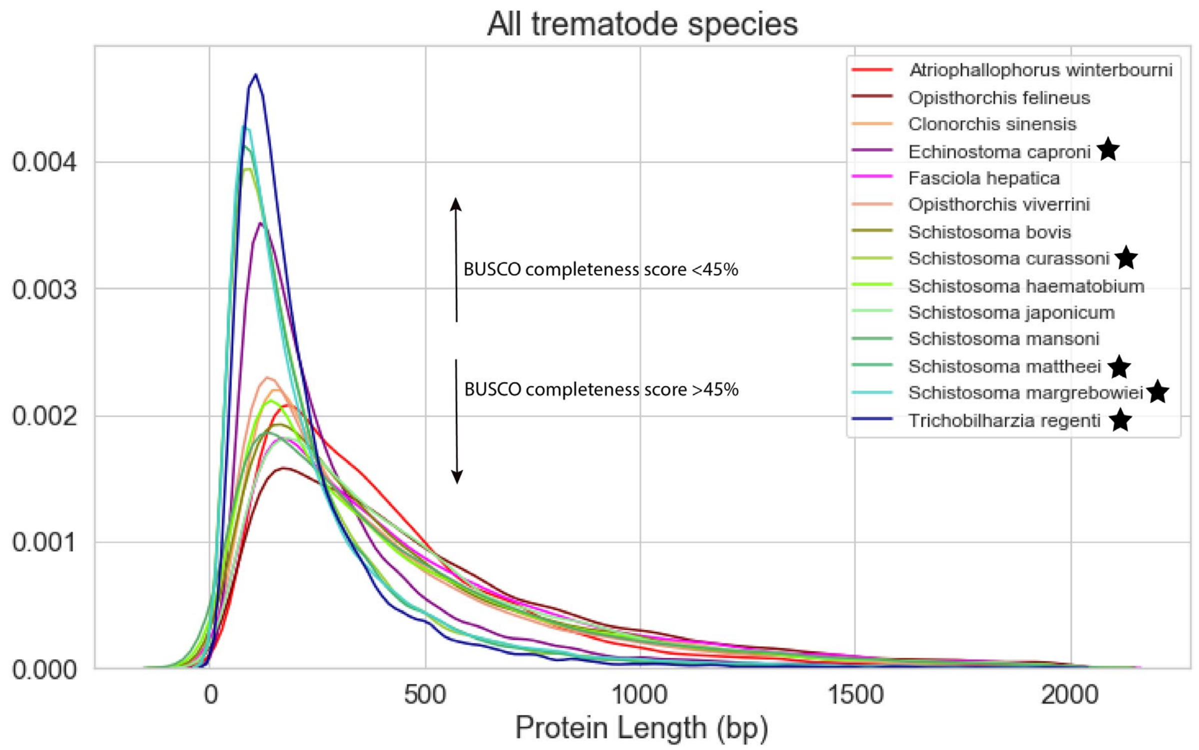

Supplementary Figure 4. Frequency distribution of the total length of proteins of 14 species of trematodes used in the analysis. The higher peaks belong to genomes with BUSCO completeness score (proportion of single copy orthologs) below 45% indicated by an arrow on the figure and by a star on the legend) and the bottom belong to genomes with BUSCO completeness score above 45%. *Atriophallophorus winterbourni* falls into the group of genomes with higher BUSCO completeness and greater number of longer proteins.

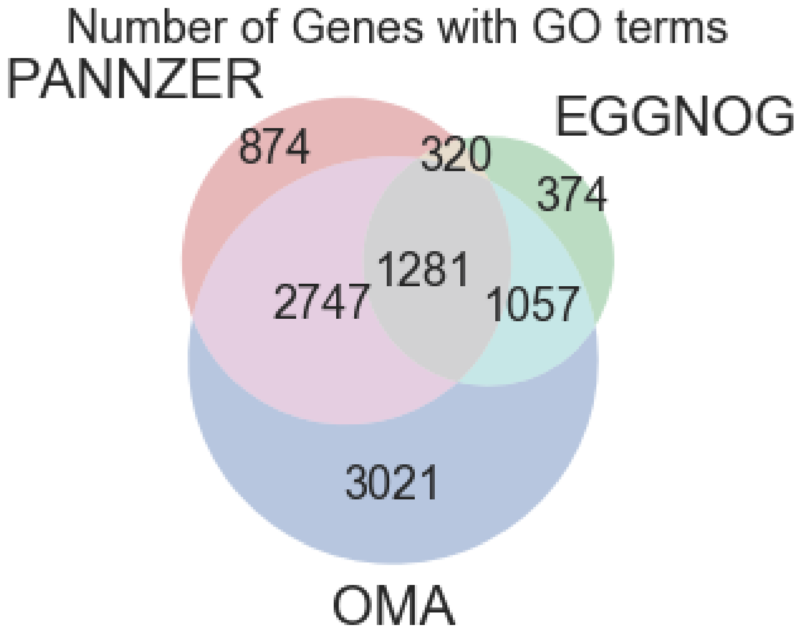

Supplementary Figure 5. The number of functionally annotated genes of *Atriophallophorus winterbourni* by Pannzer2, OMA, EggNOG and their overlap in the genes they annotated. All three programs annotated 1281 genes.

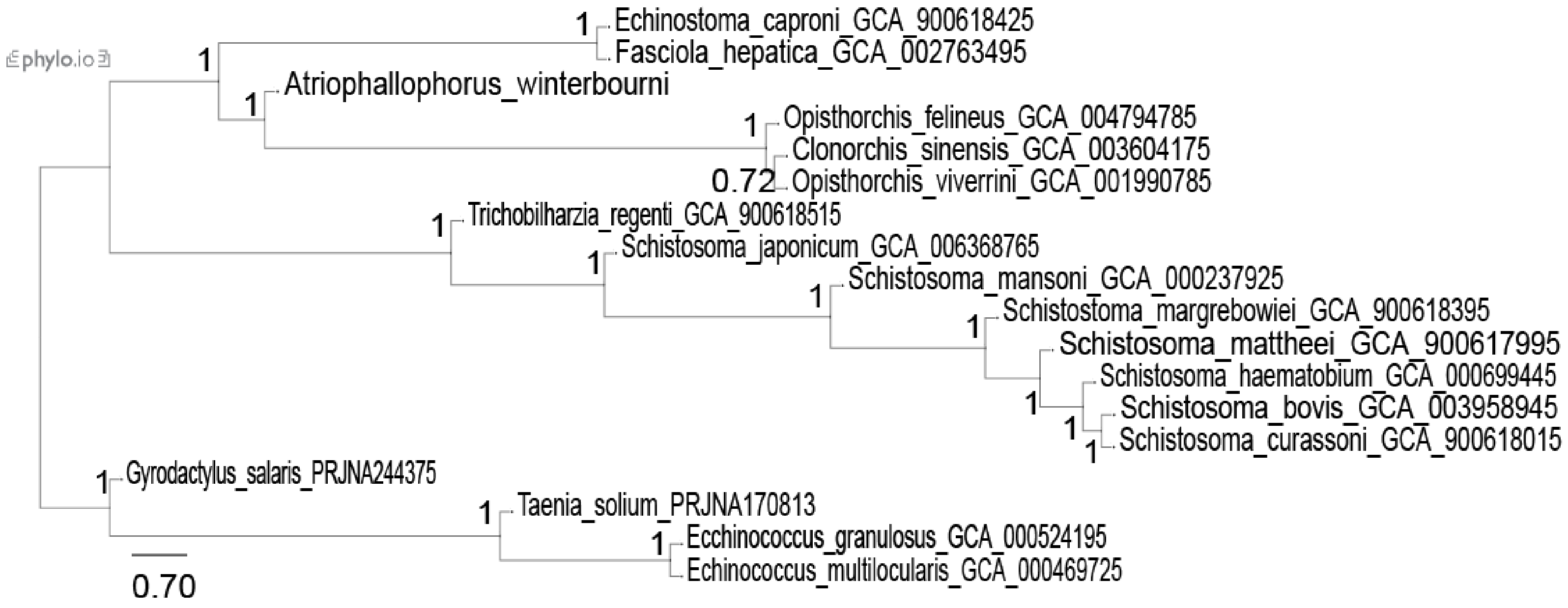

Supplementary Figure 6. Species tree created with ASTRAL III. 238 gene trees for 238 Orthologous Groups were created with MAFFT and together used in ASTRAL III to create a unifying species tree.

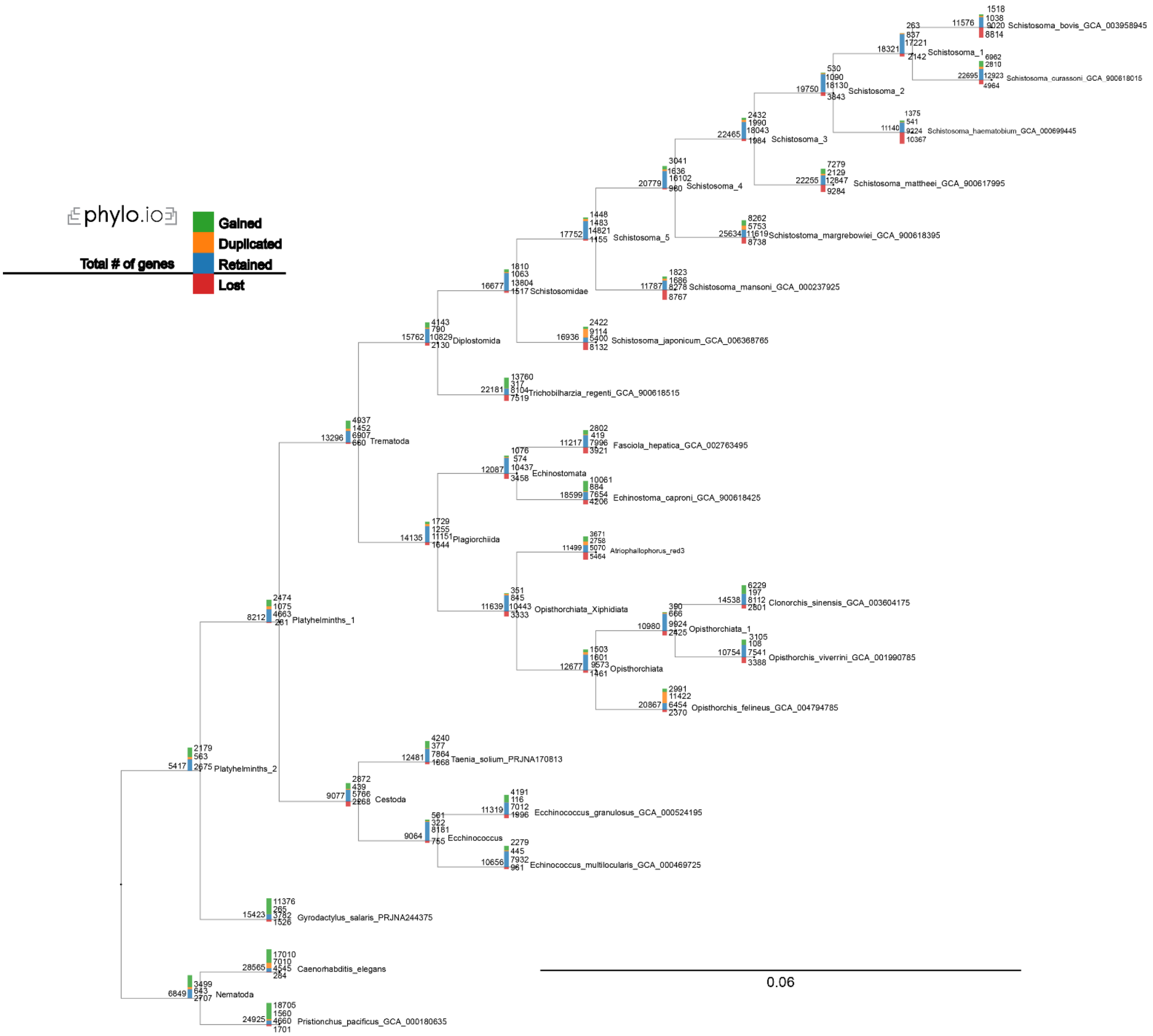

Supplementary Figure 7. Original tree created in pyHam showing total number of gained, duplicated, retained and lost genes on each branch of the phylogeny of species used in this study.

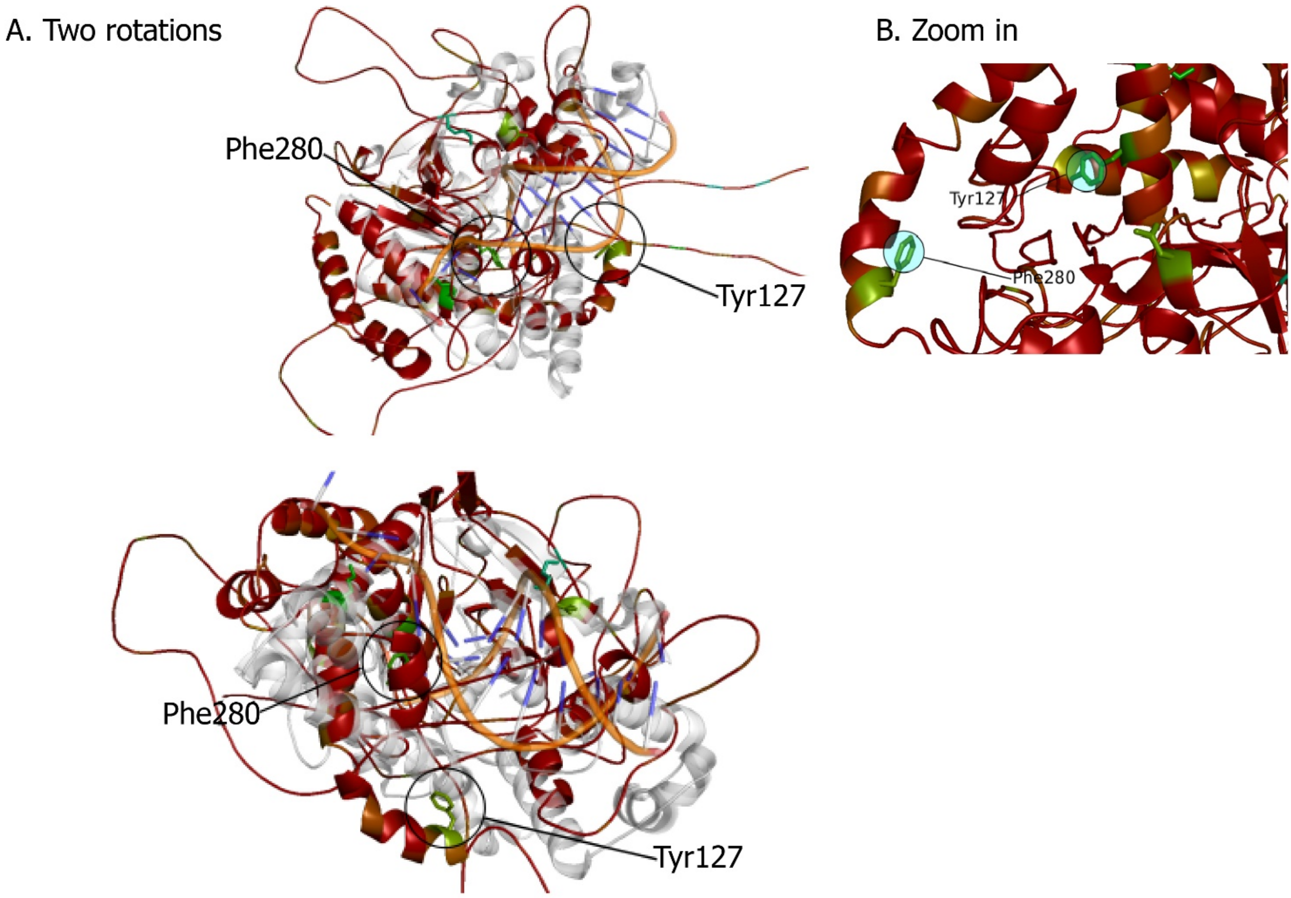

Supplementary Figure 8. **A**. A HOG 25969 PBD model (red) superimposed with DNA bound FEN1 nuclease (5V07, white) viewed from two different rotations. Green colour indicates sites with more than 90% probability of being under selection. Phe280 and Tyr127 indicate two sites are within two alpha helices which correspond to part of the typical DNA binding site present in FEN1 nucleases and show a positive selection at the site of two aromatic residues. The site numbers correspond to the MSA alignment on which the model was based and are equivalent to sites 996 (Phe280) and 1095 (Tyr127) in Table 4 for branch #1.1 **B**. A zoom-in image onto the sites under selection.

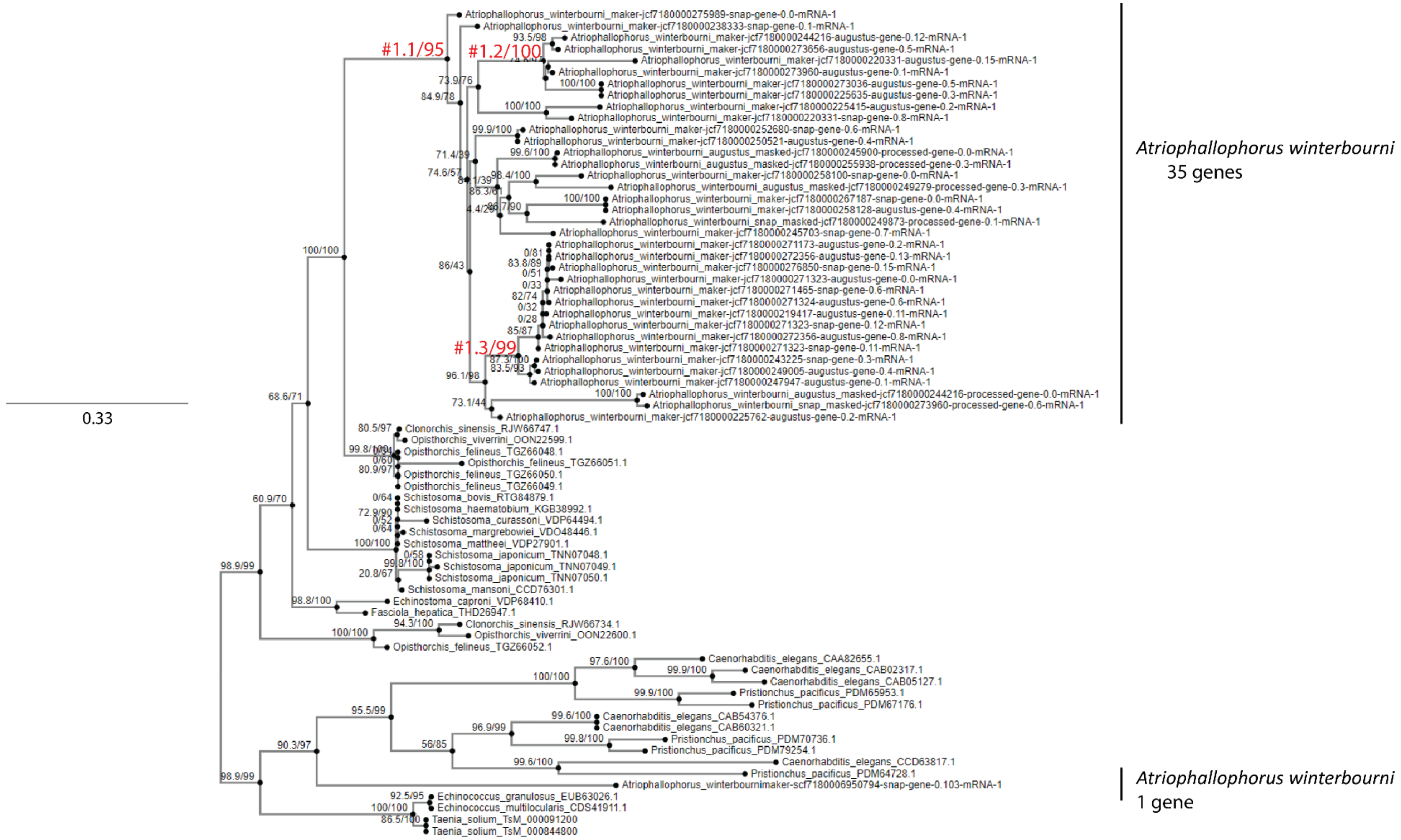

Supplementary Figure 9. Gene Tree of gene family HOG 36190 created with IQ-TREE. The tree is unrooted. Each original gene name (protein name) is followed by the species name, the names of genes of *A. winterbourni* are referring to the names in Supplementary Table 11. The numbers above branches indicate bootstrap support, for the #1 branch the bootstrap support is separated with a slash. The #1 indicated the separation between the foreground branches and the background branches (distinction used in codeml for investigation of selection). The test for selection compares the dN/dS between the foreground and the background branches.
